# c-di-GMP regulates bacterial NAD biosynthesis via targeting the transcriptional repressor NadR

**DOI:** 10.1128/mbio.01982-25

**Published:** 2025-08-18

**Authors:** Lianying Mao, Jialin Li, Xinyi Huo, Wenguang Yang, Heng Zhang, Chongyi Duan, Xihui Shen, Lei Zhang

**Affiliations:** 1 State Key Laboratory for Crop Stress Resistance and High-Efficiency Production, Shaanxi Key Laboratory of Agricultural and Environmental Microbiology, College of Life Sciences, Northwest A&F University 213667, Yangling, Shaanxi, China; Washington University in St. Louis, St. Louis, Missouri, USA

**Keywords:** c-di-GMP, NadR, NAD biosynthesis, NAD metabolism

## Abstract

As a near-ubiquitous bacterial second messenger, cyclic di-GMP (c-di-GMP) regulates a multitude of important biological processes. The regulatory effects of c-di-GMP on bacterial physiological processes are mediated through its interaction with various effector molecules, including mRNA riboswitches and proteins. Although c-di-GMP effector proteins have been widely reported, yet unknown c-di-GMP effectors in bacteria wait to be discovered, and the physiological roles of this second messenger still remain to be explored. In a c-di-GMP/transcription factor binding screen, we identified NadR, a repressor of nicotinamide adenine dinucleotide (NAD) synthesis and salvage, as a c-di-GMP-responsive transcription factor in *Salmonella enterica* serovar Typhimurium. c-di-GMP was found to bind to NadR with high affinity. c-di-GMP binding inhibits the binding of NadR to its target DNA, thus upregulating the expression of NadR-repressed genes involved in NAD synthesis and salvage. c-di-GMP also stimulates the nicotinamide mononucleotide adenylyltransferase and ribosylnicotinamide kinase activities of NadR. As a result, elevated intracellular c-di-GMP levels lead to increased NAD synthesis and enhanced resistance to DNA damage in *S*. Typhimurium. NadR proteins from three other species belonging to Enterobacterales are capable of sensing c-di-GMP, suggesting that c-di-GMP-mediated modulation of intracellular NAD homeostasis is a conserved mechanism employed by members of Enterobacterales.

**IMPORTANCE:**

Cyclic di-GMP (c-di-GMP) functions as a highly versatile signaling molecule in bacteria, orchestrating diverse physiological processes critical for survival and adaptation. While nicotinamide adenine dinucleotide (NAD) plays pivotal roles in numerous cellular processes and functions, bacteria have been shown to modulate its biosynthetic and recycling pathways through a variety of regulatory mechanisms. However, a connection between c-di-GMP signaling and NAD metabolism in bacteria has never been revealed before. Here, we identify NadR, a transcriptional repressor of NAD synthesis and salvage, as a c-di-GMP effector, and show that c-di-GMP upregulates NAD biosynthesis by activating NadR-repressed genes, thus enhancing the defense of *Salmonella* against DNA damage. Our study reveals a previously unrecognized regulatory mechanism in bacterial NAD metabolism and expands the understanding of the physiological roles of c-di-GMP in bacteria.

## INTRODUCTION

Cyclic di-GMP (c-di-GMP) is a widely distributed cyclic dinucleotide and an essential second messenger in bacteria, playing a crucial role in the regulation of bacterial behavior and environmental adaptation (1). Intracellular levels of c-di-GMP are tightly controlled by diguanylate cyclases (DGCs) containing a GGDEF domain and c-di-GMP-specific phosphodiesterases (PDEs) with an EAL or HD-GYP domain in response to internal and external signals (2). This signaling molecule allows bacteria to adapt to changing environments by interacting with a diverse array of effectors, including mRNA riboswitches and target proteins, such as transcription factors, enzymes, adapter proteins, and chaperone proteins (1, 3–5). For instance, c-di-GMP enhances biofilm formation in bacteria by modulating the synthesis of biofilm matrix components such as cellulose and amylovoran (6, 7). Additionally, c-di-GMP regulates virulence and antioxidant defense by targeting bacterial secretion systems, two-component systems, and stress response regulators (4, 8–10). Nevertheless, more c-di-GMP effectors and the physiological roles of c-di-GMP remain to be further explored.

Nicotinamide adenine dinucleotide is found in all living cells and exists as oxidized NAD^+^ and reduced NADH forms (11, 12). NAD is an indispensable redox cofactor in cells and acts as an electron carrier in innumerable redox reactions (12). Beyond its vital role in energy metabolism, NAD functions as a critical co-substrate for NAD-consuming enzymes involved in many metabolic and regulatory processes, such as sirtuins, ADP-ribosyltransferases, NAD-dependent deacetylase, and bacterial DNA ligase (13–15). In bacteria, NAD is synthesized through the *de novo* and salvage pathways (16, 17). NAD is produced *de novo* from aspartate in most bacteria and from tryptophan in a few species, while some bacteria have evolved various salvage pathways to produce NAD by recycling its precursor metabolites, including nicotinamide (Nam), nicotinic acid, and ribosyl nicotinamide (RNam) (16, 18, 19). In many bacteria, including *Salmonella*, NAD is synthesized *de novo* from aspartate in five sequential enzymatic reactions catalyzed by L-aspartate oxidase NadB, quinolinate synthetase NadA, quinolinate phosphorybosyltransferase NadC, nicotinate mononucleotide (NaMN) adenylyltransferase NadD, and NAD synthetase NadE (16, 20). A two-step conversion of Nam to NaMN by nicotinamidase PncA and nicotinate phosphoribosyltransferase PncB represents the most widely distributed salvage pathway for NAD biosynthesis, whereas another salvage pathway includes the RNam uptake via its transporter PnuC and its phosphorylation by the multifunctional NadR protein (14, 16, 21).

Depletion of the NAD pool and accumulation of some intermediates are both harmful to the cell, and thus, NAD homeostasis is tightly regulated (22). To date, four types of transcription factors, including NadR, NiaR, NrtR, and NadQ, have been identified as repressors of NAD synthesis and/or salvage genes (23–26). The NadR protein of *Salmonella* and *Escherichia coli* was found to function as a transcriptional repressor of certain NAD synthesis and salvage genes such as *nadB*, *nadA*, *pnuC,* and *pncB* in response to intracellular NAD and ATP levels (23, 27). The repressor function of NadR is provided by an N-terminal helix-turn-helix (HTH) domain, followed by two domains with nicotinamide mononucleotide (NMN) adenylyltransferase (NMN-AT) and RNam kinase (RNam-K) activities, respectively (27, 28). However, the occurrence of the trifunctional NadR protein is restricted to members of the order Enterobacterales, and most NadR orthologs in bacterial lineages beyond Enterobacterales lack the N-terminal DNA-binding domain and thus function only as salvage enzymes but not as transcriptional regulators (16, 20, 29–31). While all three activities of NadR in *Salmonella* have been shown to be feedback regulated by NAD in conjunction with ATP (27), it remains unclear whether other signals regulate NAD biosynthesis via this transcription factor.

In this study, we set out to screen for new c-di-GMP receptors in a *Salmonella* Typhimurium transcription factor library and identified NadR as a previously unidentified c-di-GMP effector. We found that c-di-GMP binding inhibits the binding of NadR to its target promoters, thus upregulating the expression of NadR-repressed NAD synthesis and salvage genes such as *nadB*, *nadA,* and *pnuC*. c-di-GMP was also shown to stimulate the NMN-AT and RNam-K activities of NadR. We further found that an increase in intracellular c-di-GMP levels upregulates NAD synthesis via targeting NadR, thus increasing the resistance of *S*. Typhimurium against DNA damage. Our results also suggest that sensing of c-di-GMP by NadR represents a general mechanism employed by members of Enterobacterales to regulate NAD biosynthesis, expanding our understanding of the physiological roles of c-di-GMP in bacteria.

## RESULTS

### Identification of NadR as a candidate c-di-GMP receptor protein in *S*. Typhimurium

**Fig 1:**
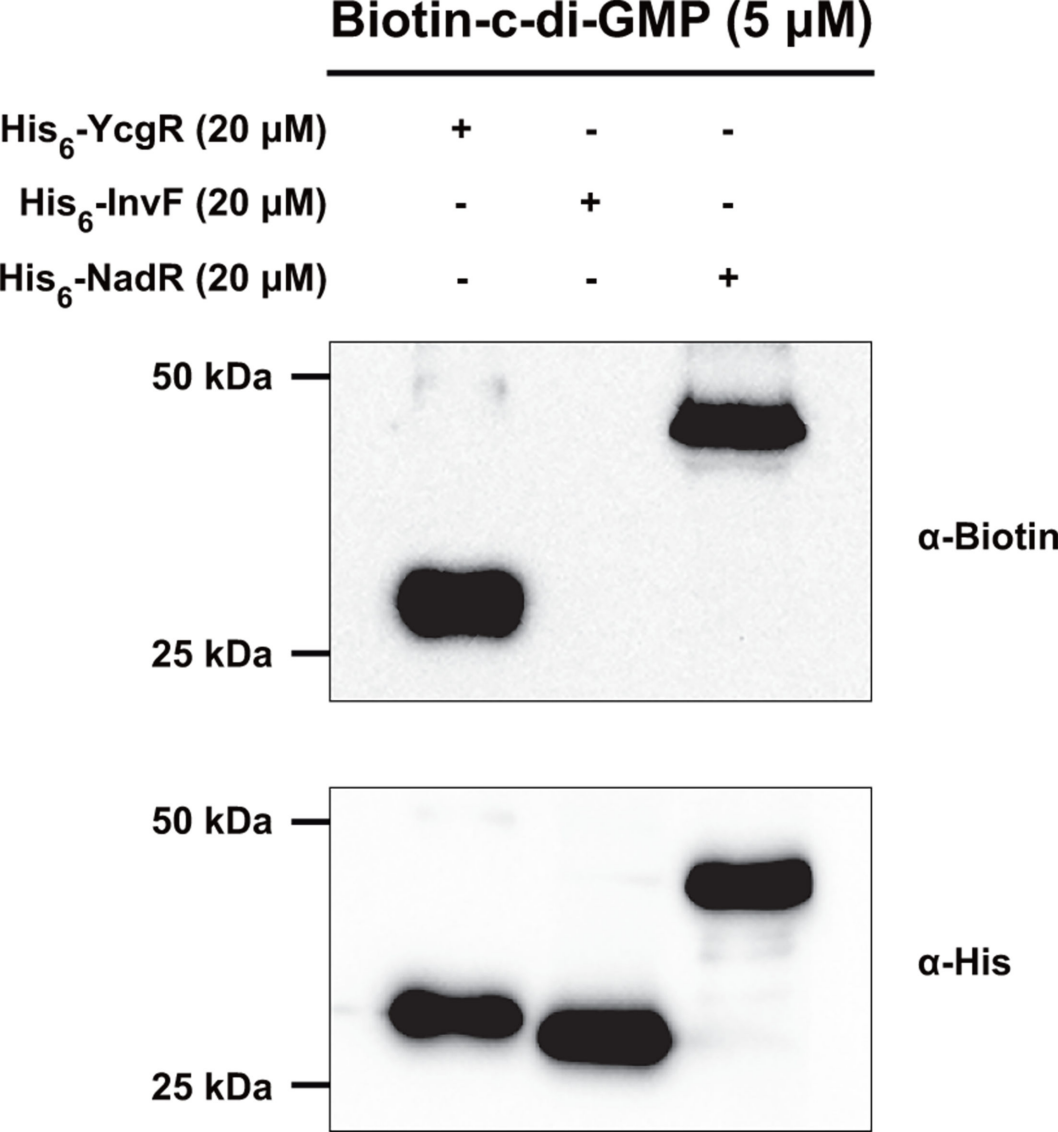
Identification of NadR as a c-di-GMP receptor protein in *S*. Typhimurium. The UV-crosslinking assays detecting the binding of His_6_-NadR to biotinylated c-di-GMP. His_6_-YcgR was used as a positive control, and His_6_-InvF was used as a negative control. The reaction samples were separated by SDS-PAGE, transferred to nitrocellulose membrane, and probed with the anti-biotin antibody streptavidin horseradish peroxidase (top). The amount of each protein transferred to the membrane was also determined by western blot using the anti-His antibody (bottom). The blots presented are representative of three independent experiments with similar results.

Transcription factors are one of the most common types of c-di-GMP effectors in bacteria (10, 32, 33). To identify regulatory proteins that sense c-di-GMP in *S*. Typhimurium, all genes encoding proteins from the genome (GenBank accession number: GCA_000006945.2) were functionally annotated in the NCBI non-redundant database, and 195 genes predicted to encode transcription factors were picked out based on the functional annotations. With the exception of the gene encoding the identified c-di-GMP-responsive transcription factor H-NS (5), 194 predicted regulatory genes were cloned into pET-28a with an N-terminal His_6_ tag. The *Escherichia coli* BL21(DE3) strain carrying recombinant plasmids was divided into 39 groups, and the recombinant proteins were co-expressed and co-purified. The anti-His tag staining showed that 163 His_6_-tagged proteins were successfully expressed (Fig. S1A; Table S1). We then screened transcriptional regulatory proteins that can interact with c-di-GMP by an ultraviolet (UV)-crosslinking assay using biotin-labeled c-di-GMP. YcgR, a well-known c-di-GMP effector, is used as a positive control. We found a strong c-di-GMP-binding signal in a group containing six recombinant proteins (Fig. S1B). However, among the 163 successfully expressed regulatory proteins, the c-di-GMP-binding signal was not detected in other groups (Fig. S1B). Every protein of the group with a c-di-GMP-binding signal was separately purified for further c-di-GMP binding screen, and NadR (STM4580) was found to be a candidate regulator that may bind c-di-GMP (Fig. S1C). YcgR could obviously bind biotinylated c-di-GMP, while InvF, which was used as a negative control, showed no biotin-binding signal (Fig. 1). Under the same experimental conditions, NadR was confirmed to bind to c-di-GMP, as proven by a specific and strong biotin-binding signal observed on the gel (Fig. 1).

### c-di-GMP binds to NadR with high affinity

**Fig 2:**
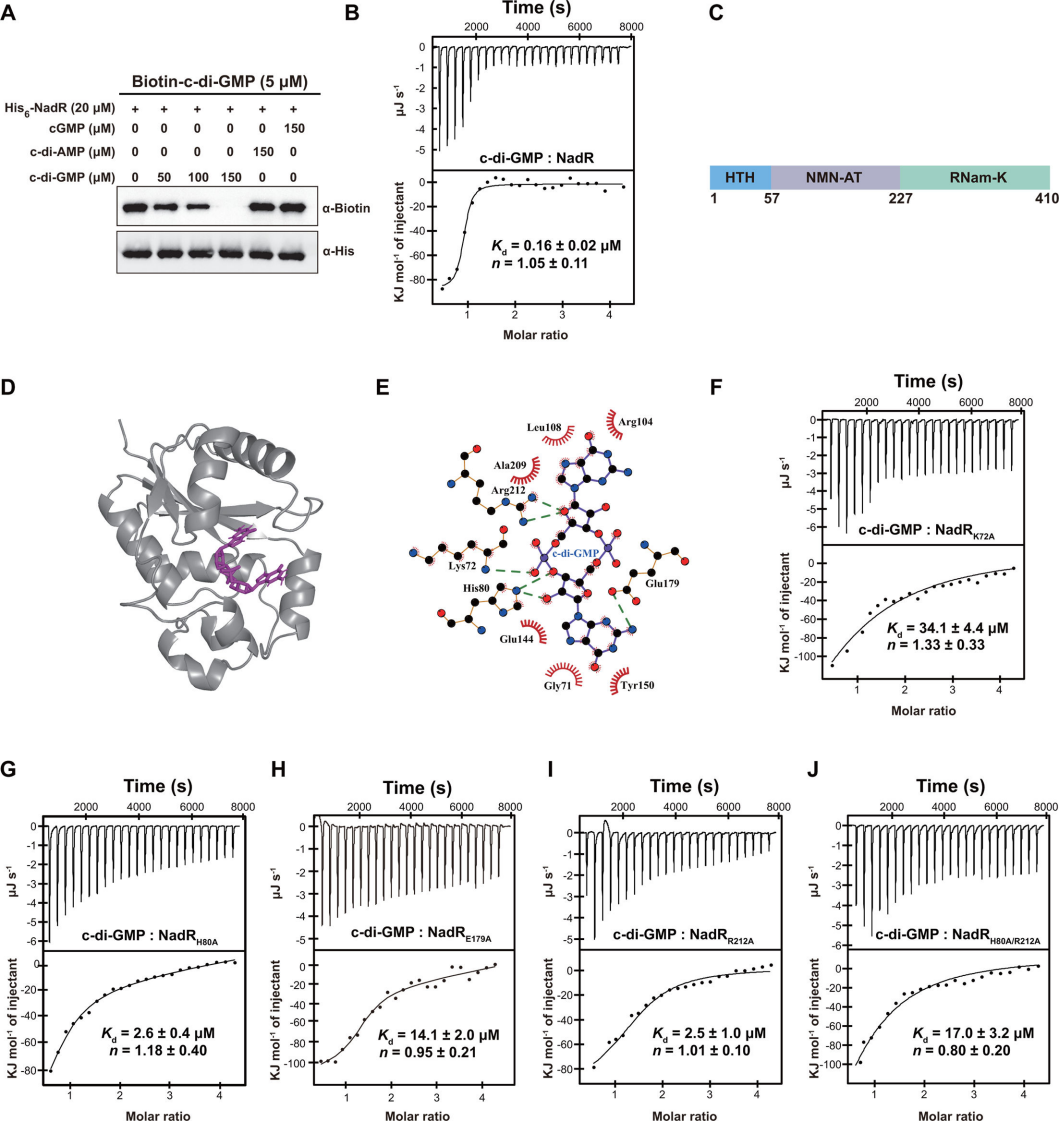
c-di-GMP binds with high affinity and specificity to the NadR protein in *S*. Typhimurium. (**A**) The UV-crosslinking assays for binding specificity of His_6_-NadR to biotinylated c-di-GMP. Competition experiments were performed by the addition of unlabeled c-di-GMP, c-di-AMP, or cGMP simultaneously with biotinylated c-di-GMP to the reaction mixtures. (**B**) ITC assays for the interaction between NadR and c-di-GMP. The original titration data (the upper plot) and integrated heat measurements (the lower plot) are shown. The solid line in the lower plot represents the best fit to a one-site binding model of the interaction of NadR with c-di-GMP. (**C**) Schematic illustrating the domain organization of NadR. The HTH DNA-binding domain (residues 1–57), the NMN-AT domain (residues 58–227), and the RNam-K domain (residues 228–410) are indicated. (**D**) Predicted binding mode of c-di-GMP to the NMN-AT domain of NadR. (**E**) Schematic of the predicted contacts between c-di-GMP and NadR. Potential hydrogen bonds are indicated as green dashed lines. (**F–J**) ITC assays for the interactions of the NadR variants K72A (**F**), H80A (**G**), E197A (**H**), R212A (**I**), or H80A/R212A (**J**) with c-di-GMP. (**A, B, and F–J**) Data shown are representatives of three independent experiments with similar results. (**B and F–J**) *K*_*d*_ and complex stoichiometry (***N***) are presented as mean ± SD of three independent experiments.

Next, we further determined the binding specificity and affinity between NadR and c-di-GMP. In the UV-crosslinking assay, we observed that the addition of unlabeled c-di-GMP could inhibit the binding of biotinylated c-di-GMP to NadR in a concentration-dependent manner (Fig. 2A). In contrast, the addition of unlabeled cGMP or c-di-AMP at a dose corresponding to the highest level of c-di-GMP did not result in an observable weakening in the binding of biotinylated c-di-GMP to NadR (Fig. 2A), indicating that NadR specifically binds c-di-GMP, but not cGMP or c-di-AMP. Furthermore, isothermal titration calorimetry (ITC) analysis showed that c-di-GMP binds to NadR at a 1:1 stoichiometry with a binding affinity (*K*_*d*_) of 0.16 ± 0.02 µM (Fig. 2B), which is comparable to previously reported *K*_*d*_ values for other well-established c-di-GMP receptors (4, 5, 34). In contrast, interactions of NadR with cGMP or c-di-AMP were not detected by ITC analysis under the same experimental conditions (Fig. S2A and B). Together, these results confirmed the binding specificity and high affinity of NadR with c-di-GMP.

The NadR protein in *S*. Typhimurium has been reported to contain three functional domains: an N-terminal HTH DNA-binding domain, the central NMN-AT domain, and a C-terminal RNam-K domain (Fig. 2C) (27). A docking simulation was performed by AutoDock Vina 1.1.2 between the 3D structure of NadR predicted by the AlphaFold Protein Structure Database (AFDB accession number: AF-P24518-F1; https://alphafold.ebi.ac.uk/) and c-di-GMP. The conformation with the lowest binding energy of −9.5 kcal mol^−1^ (Fig. 2D) suggests that c-di-GMP makes close contact with G71, K72, H80, R104, L108, E144, Y150, E179, A209, and R212 in the NMN-AT domain of NadR (Fig. 2E). Alanine substitution mutation of K72 or E179 resulted in an 88- to 213-fold decrease in the c-di-GMP-binding affinity for NadR, while mutation of H80 or R212 resulted in a more than 15-fold decrease in the c-di-GMP-binding affinity (Fig. 2F through I). Furthermore, the double mutant H80A/R212A showed a more than 100-fold decrease in the c-di-GMP-binding affinity compared to wild-type NadR (Fig. 2J). These results indicate that c-di-GMP binds to the NMN-AT domain of NadR, and the four residues K72, H80, E179, and R212 are important for the specific interaction between NadR and c-di-GMP.

### c-di-GMP inhibits the binding of NadR to its target promoters but enhances the NMN-AT and RNam-K activities of NadR

**Fig 3:**
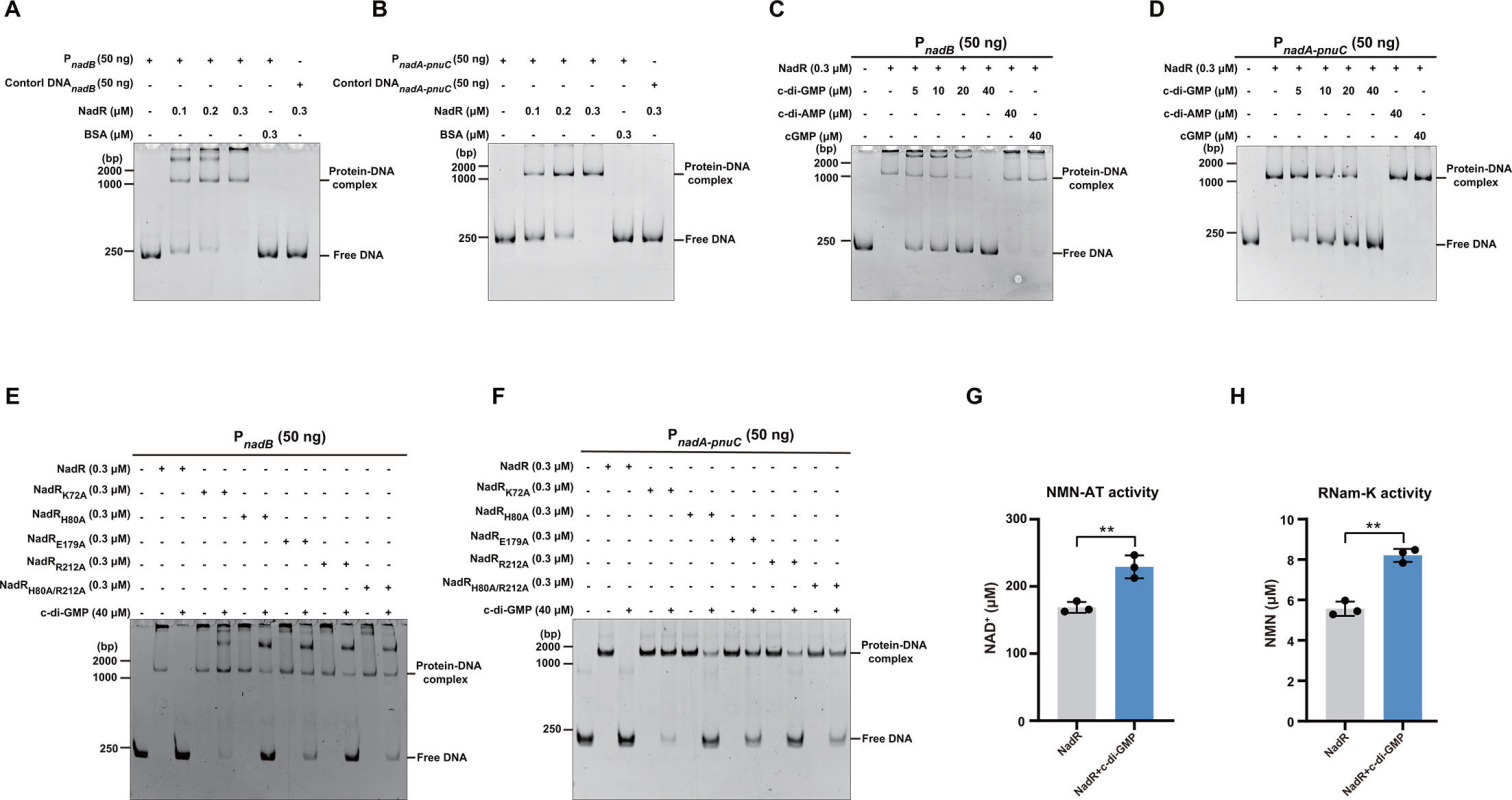
c-di-GMP interferes with NadR binding to its target promoters but stimulates its NMN-AT and RNam-K activities. (**A and B**) EMSAs demonstrating specific binding of NadR to promoters of the *nadB* gene (**A**) and the *nadA-pnuC* operon (**B**). DNA fragments amplified from the coding regions of *nadB* (**A**) and *nadA* (**B**) were used as DNA negative controls, and BSA was used as the protein control. (**C and D**) EMSAs for NadR binding to the promoters of *nadB* (**C**) and *nadA-pnuC* (**D**) in the absence or presence of nucleotides. Nucleotides were added simultaneously with NadR to the reaction system. (**E and F**) EMSAs for the binding of NadR and its variants K72A, H80A, E179A, R212A, and H80A/R212A to the promoters of *nadB* (**E**) and *nadA-pnuC* (**F**) in the absence or presence of c-di-GMP. (**G and H**) c-di-GMP enhances the NMN-AT (**G**) and RNam-K (**H**) activities of NadR. (**A–F**) Gels shown are representative of three independent experiments with similar results. (**G and H**) Data are mean ± SD of three biological replicates. Two-sided, unpaired Student’s *t*-test was used for statistical analyses. **P < 0.01.

NadR has been shown to be a transcriptional repressor that represses the expression of the NAD *de novo* biosynthetic genes *nadB* and *nadA* and the salvage genes *pnuC* and *pncB* (28, 35, 36). Consistent with these studies, our electrophoretic mobility shift assays (EMSAs) showed that NadR specifically binds to the promoter sequences of the *nadB* gene as well as the *nadA-pnuC* operon (Fig. 3A and B). We then investigated whether c-di-GMP affects the binding of NadR to the two target promoters. When c-di-GMP was added together with NadR, this second messenger decreased the formation of the NadR-DNA complexes in a concentration-dependent manner (Fig. 3C and D). In contrast, the addition of both cGMP and c-di-AMP at a dose corresponding to the highest level of c-di-GMP was unable to impair the NadR-DNA complex formation (Fig. 3C and D). These results indicate that the binding of c-di-GMP to NadR inhibits its DNA binding activity.

While the c-di-GMP binding site is outside the DNA binding domain (Fig. 2C through E), we found that the K72A, H80A, E179A, R212A, and H80A/R212A mutations of NadR did not result in an observable weakening in its binding to the two target promoters (Fig. 3E and F). While c-di-GMP completely abrogated the formation of NadR-DNA complexes, the binding of the NadR variants K72A, E179A, and H80A/R212A to the two target promoters was only slightly impaired by the same concentration of c-di-GMP (Fig. 3E and F). Nevertheless, the binding of the NadR variants H80A and R212A was largely abrogated by the addition of c-di-GMP, which is consistent with the ITC data that the H80A and R212A variants still retain a relatively high binding affinity for c-di-GMP (Fig. 2G and I). These results suggest that the K72A, E179A, and H80A/R212A mutations leave the DNA binding activity of NadR unaffected but drastically impair its ability to bind and respond to c-di-GMP.

NAD has been shown to inhibit the NMN-AT and RNam-K activities of NadR (27). We thus investigated whether c-di-GMP binding affects the two enzymatic activities of NadR. *In vitro* enzymatic activity assays in the presence and absence of c-di-GMP followed by HPLC analysis showed that c-di-GMP enhances both enzymatic activities of NadR (Fig. 3G and H). Together, our results suggest that binding of c-di-GMP to NadR inhibits its DNA binding activity while stimulating its NMN-AT and RNam-K activities.

### c-di-GMP, ATP, and NAD competitively bind to NadR

The DNA binding activity of NadR has been reported to be inhibited by ATP, while high levels of NAD can compete with ATP for the same binding site to restore the DNA binding activity of NadR (27). A previous study has suggested that R212 is an ATP-binding residue of NadR, and the R212C mutation prevents ATP from binding to the central NMN-AT domain (27). Our docking analysis and ITC experiments also showed that R212 is a key residue for c-di-GMP binding (Fig. 2E and I), suggesting that ATP and c-di-GMP have partially overlapping binding sites on NadR. To investigate the potential overlapping binding sites of ATP, NAD, and c-di-GMP on NadR, we also performed molecular docking to analyze the interactions of NadR with ATP and NAD. The best docking conformations, which exhibit the lowest binding energies of −9.6 and −10.1 kcal mol^−1^ for ATP and NAD, respectively (Fig. S3A and B), were selected for ligand binding analysis using LigPlot^+^ (37) (Fig. S3C and D). The three conformations suggest that all three ligands, ATP, NAD, and c-di-GMP, form hydrogen bonds with the residues K72, H80, and R212 of NadR (Fig. 2E; Fig. S3C and D). Indeed, the mutation of K72 to alanine led to 8- to 41-fold reductions in the binding affinities for ATP and NAD (Fig. S4A through D). By contrast, the E179A mutation of NadR did not significantly affect its binding affinities for ATP and NAD (Fig. S4E and F), indicating that the residue E179, which is required for c-di-GMP binding, does not participate in interactions with ATP or NAD. These results indicate that ATP, NAD, and c-di-GMP have partially overlapping binding sites on NadR.

**Fig 4:**
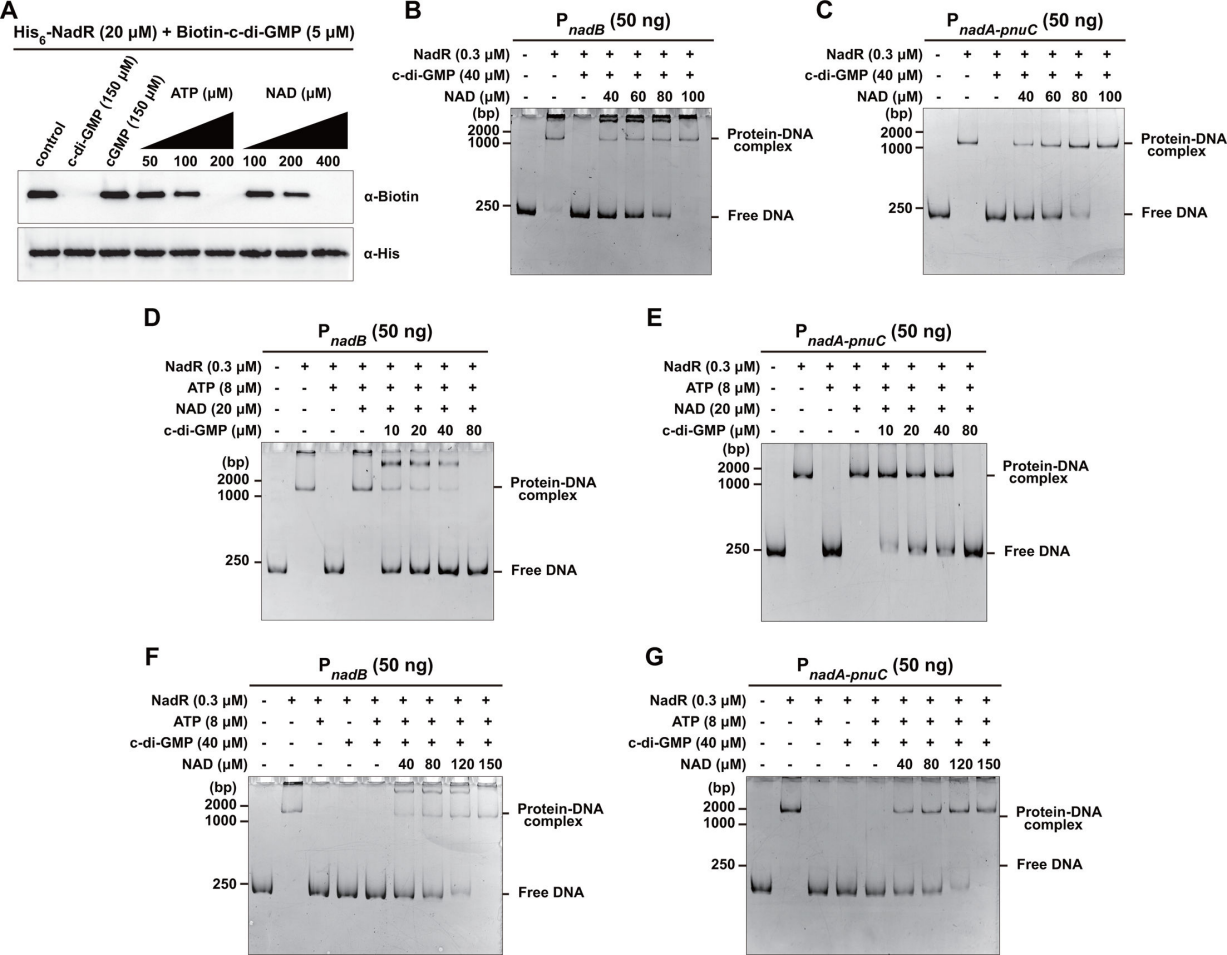
c-di-GMP, ATP, and NAD can competitively bind to NadR. (**A**) The UV-crosslinking assay showing binding of His_6_-NadR to biotinylated c-di-GMP in the absence or presence of unlabeled nucleotides or NAD. The unlabeled nucleotides or NAD were added simultaneously with biotinylated c-di-GMP to the reaction system containing His_6_-NadR. (**B and C**) EMSAs for NadR binding to the promoters of *nadB* (**B**) and *nadA-pnuC* (**C**) in the absence or presence of c-di-GMP and/or different concentrations of NAD. (**D and E**) EMSAs for the binding of NadR to the promoters of *nadB* (**D**) and *nadA-pnuC* (**E**) in the absence or presence of ATP, NAD, and/or different concentrations of c-di-GMP. (**F and G**) EMSAs for the binding of NadR to the promoters of *nadB* (**F**) and *nadA-pnuC* (**G**) in the absence or presence of ATP and c-di-GMP and/or different concentrations of NAD. (**A–G**) Blots and gels shown are representative of three independent experiments with similar results. (**B–G**) c-di-GMP, ATP, or NAD were added simultaneously with NadR to the reaction system.

As expected, we observed that the addition of unlabeled ATP and NAD could inhibit the binding of biotinylated c-di-GMP to NadR in a concentration-dependent manner in the UV-crosslinking assay (Fig. 4A), supporting that c-di-GMP, ATP, and NAD competitively bind to NadR. Consistent with previous studies (27), the EMSA results showed that ATP interferes with the binding of NadR to the promoters of *nadB* and the *nadA-pnuC* operon (Fig. S5A and B), whereas the ATP-mediated inhibition of NadR binding to DNA is reversed by NAD (Fig. S5C and D). Furthermore, c-di-GMP-mediated inhibition of NadR binding to DNA was also reversed by increasing concentrations of NAD (Fig. 4B and C). Nevertheless, when a relatively high level of NAD allowed NadR to bind DNA in the presence of ATP, the addition of c-di-GMP reduced the formation of NadR-DNA complexes in a concentration-dependent manner (Fig. 4D and E), indicating that c-di-GMP can also compete with NAD for binding to NadR and thus inhibit the binding of NadR to its target promoters. In addition, when both ATP and c-di-GMP were added simultaneously to inhibit the binding of NadR to DNA, the addition of NAD also restored NadR-DNA binding in a dose-dependent fashion (Fig. 4F and G). Together, these results suggest that c-di-GMP, ATP, and NAD can competitively bind to NadR, with ATP and c-di-GMP inhibiting the DNA binding activity of NadR, while a high level of NAD allows NadR to bind DNA in the presence of ATP and c-di-GMP.

### c-di-GMP regulates the expression of NAD synthesis and salvage genes via targeting NadR

**Fig 5:**
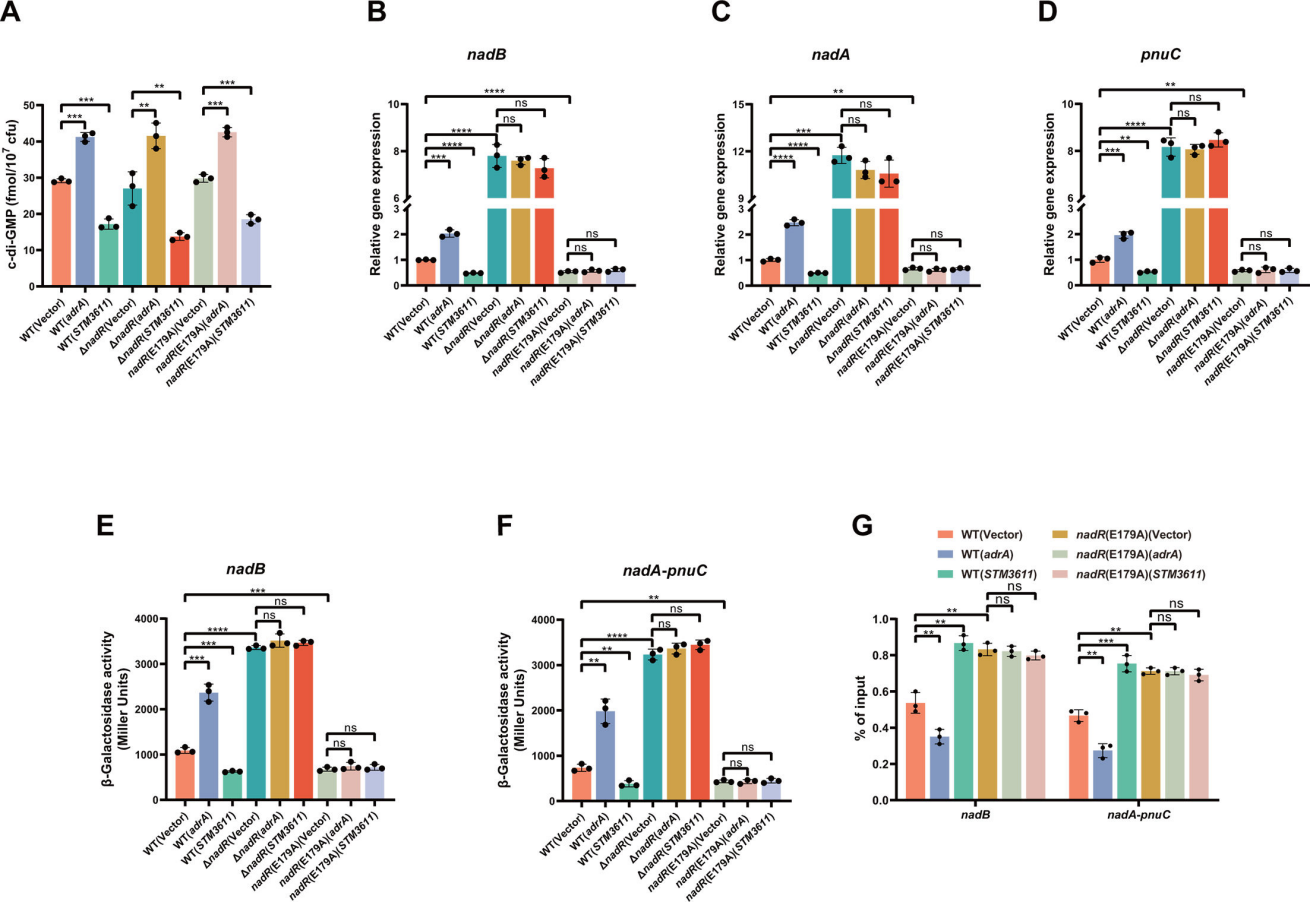
Changes in intracellular c-di-GMP levels regulate the expression of genes involved in the *de novo* and salvage pathways of NAD biosynthesis via targeting NadR. (**A**) Intracellular c-di-GMP levels in the wild-type strain, the Δ*nadR* mutant, and the point mutant *nadR*(E179A) were modulated by overexpression of *adrA* or *STM3611*. (**B–D**) qRT-PCR analysis of the mRNA levels of *nadB* (**B**), *nadA* (**C**), and *pnuC *(D) in the wild-type strain, the Δ*nadR* mutant, the point mutant *nadR *(E179A), and their derivatives overexpressing *adrA* or *STM3611*. Expression levels were normalized to 16S rRNA and presented as values relative to that of the wild type without gene overexpression. (**E and F**) The promoter activities of the *nadB* gene (**E**) and the *nadA-pnuC* operon (**F**) in the wild type, Δ*nadR*, *nadR*(E179A), and their derivatives overexpressing *adrA* or *STM3611*. (**G**) Chromatin immunoprecipitation (ChIP)-qPCR quantifying binding of HA-NadR and HA-NadR_E179A_ at the promoters of *nadB* and *nadA-pnuC* in the wild type, *nadR*(E179A), and their derivatives overexpressing *adrA* or *STM3611*. The ChIP-qPCR signals were normalized to their respective DNA inputs. (**A–G**) Data are mean ± SD of three biological replicates. Two-sided, unpaired Student’s *t*-test was used for statistical analyses. ns, not significant; **P < 0.01; ***P < 0.001; and ****P < 0.0001.

The EMSA results showed that the binding of c-di-GMP to NadR inhibits the DNA binding activity of NadR (Fig. 3C and D), leading us to speculate that changes in intracellular c-di-GMP levels will affect the expression of NAD *de novo* synthesis and salvage genes through NadR. Our previous study has shown that overexpression of *adrA* (*STM0385*), which encodes a DGC, can lead to an increase in intracellular c-di-GMP levels, whereas overexpression of *STM3611,* which encodes a PDE, can lead to a decrease in intracellular c-di-GMP levels in *S*. Typhimurium (5). Thus, we overexpressed *adrA* or *STM3611* in the wild-type strain, the Δ*nadR* mutant, and the point mutant *nadR*(E179A). As expected, overexpression of *adrA* or *STM3611* resulted in similar changes in intracellular c-di-GMP levels in Δ*nadR* and *nadR*(E179A) compared to the wild-type strain (Fig. 5A). Quantitative real-time PCR (qRT-PCR) analysis showed that *adrA* overexpression significantly upregulated, whereas *STM3611* overexpression significantly downregulated, the mRNA levels of the *nadB*, *nadA,* and *pnuC* genes in the wild type (Fig. 5B through D). While the mRNA levels of the *nadB*, *nadA,* and *pnuC* genes in Δ*nadR* were 8- to 10-fold increased compared to the wild type, overexpression of *adrA* or *STM3611* in Δ*nadR* resulted in no significant difference in the expression levels of these NAD synthesis and salvage genes (Fig. 5B through D). Moreover, the *nadR*(E179A) mutant showed significantly reduced expression of *nadB*, *nadA,* and *pnuC* compared to the wild type, whereas the mRNA levels of these genes were not significantly changed by overexpression of *adrA* or *STM3611* in the point mutant (Fig. 5B through D), suggesting that the E179A mutation of NadR abrogates its response to intracellular c-di-GMP fluctuations. Promoter-reporter assays also showed that the promoter activities of *nadB* and the *nadA-pnuC* operon in the wild type were significantly increased following the overexpression of *adrA* but were significantly inhibited following the overexpression of *STM3611,* whereas overexpression of *adrA* or *STM3611* did not significantly change the activities of the two promoters in Δ*nadR* and *nadR*(E179A) (Fig. 5E and F). Collectively, these results indicate that c-di-GMP positively regulates the expression of the NAD *de novo* synthesis genes *nadB* and *nadA* and the salvage gene *pnuC* at the transcriptional level through NadR.

To further evaluate the effects of c-di-GMP on NadR binding to DNA in cells, chromatin immunoprecipitation (ChIP) assays were conducted using an anti-HA antibody on *S*. Typhimurium strains expressing *in situ* HA-tagged NadR and NadR_E179A_. The ChIP-quantitative PCR (ChIP-qPCR) results showed that the overexpression of *adrA* in the wild type led to a notable decrease in HA-NadR enrichment at the promoter regions of *nadB* and the *nadA-pnuC* operon*,* while the overexpression of *STM3611* resulted in a significant increase in its enrichment at these two promoters (Fig. 5G). Furthermore, the E179A mutation of HA-NadR significantly increased its occupancy at the two promoter regions, whereas the binding of HA-NadR_E179A_ to the two promoters was not significantly affected by overexpression of *adrA* or *STM3611* (Fig. 5G), confirming that the E179A mutant of NadR loses response to c-di-GMP *in vivo*. Together, these results indicate that binding of c-di-GMP to NadR inhibits the DNA-binding activity of NadR, consequently leading to upregulation of NadR-repressed genes, such as *nadB*, *nadA,* and *pnuC* in *S*. Typhimurium.

### Elevated c-di-GMP levels stimulate NAD synthesis and enhance DNA damage resistance via targeting NadR in *S*. Typhimurium

**Fig 6:**
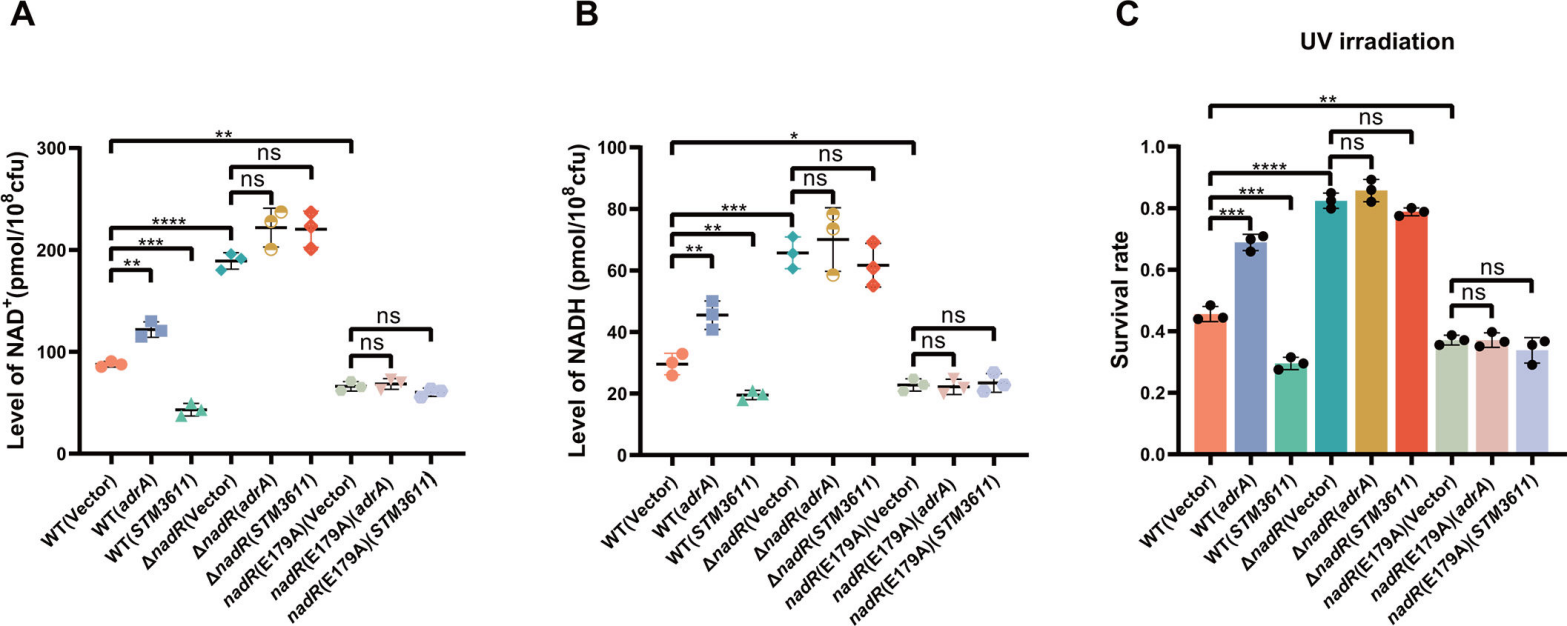
Changes in intracellular c-di-GMP levels modulate NAD biosynthesis via targeting NadR and enhance DNA damage resistance of *S*. Typhimurium. (**A and B**) Comparison of the intracellular level of NAD^+^ (**A**) and NADH (**B**) among the wild type, Δ*nadR*, *nadR*(E179A), and their derivatives overexpressing *adrA* or *STM3611*. (**C**) Survival rates of the wild type, Δ*nadR*, *nadR*(E179A), and their derivatives overexpressing *adrA* or *STM3611* after exposure to UV irradiation. The data are shown as means ± SD of three biological replicates. Student’s *t*-test was used for statistical analyses. ns, not significant; *P < 0.05; **P < 0.01; ***P < 0.001; and ****P < 0.0001.

Our *in vitro* and *in vivo* results demonstrated that c-di-GMP binds to the NadR protein to inhibit its binding to the promoters of its target genes, and thus, changes in intracellular c-di-GMP levels will lead to altered expression of the NadR-repressed genes involved in NAD *de novo* synthesis and salvage (Fig. 3 to 5). Consistently, overexpression of *adrA* in the wild-type strain significantly increased the levels of intracellular NAD^+^ and NADH, whereas overexpression of *STM3611* in this strain led to significantly reduced intracellular concentrations of NAD^+^ and NADH (Fig. 6A and B). As expected, intracellular levels of NAD^+^ and NADH were higher in the Δ*nadR* mutant compared to the wild type, whereas there were no significant differences in the levels of NAD^+^ and NADH between Δ*nadR* and its derivatives overexpressing *adrA* or *STM3611* (Fig. 6A and B). Moreover, the *nadR*(E179A) mutant showed reduced levels of NAD^+^ and NADH compared to the wild type, while overexpression of *adrA* or *STM3611* in *nadR*(E179A) resulted in no significant changes in intracellular levels of NAD^+^ and NADH (Fig. 6A and B). Therefore, these results indicate that fluctuations in intracellular c-di-GMP levels can regulate NAD biosynthesis via targeting NadR, suggesting an important role of c-di-GMP in maintaining the homeostasis of the NAD^+^/NADH pool in *S*. Typhimurium.

While acting as a key player in various cellular processes, NAD is a critical coenzyme participating in the regulation of DNA repair (14, 15, 38). We thus examined whether c-di-GMP-mediated regulation of NAD synthesis affects DNA damage resistance in *S*. Typhimurium. Indeed, overexpression of *adrA* and *STM3611* significantly increased and decreased, respectively, the resistance to UV irradiation in the wild-type strain (Fig. 6C). When compared to the wild type, the Δ*nadR* mutant showed remarkably increased UV resistance, whereas the *nadR*(E179A) mutant showed significantly decreased UV resistance (Fig. 6C). Furthermore, overexpression of *adrA* or *STM3611* led to no significant changes in the UV resistance of Δ*nadR* and *nadR*(E179A) (Fig. 6C). These results suggest that fluctuations in intracellular c-di-GMP levels regulate DNA damage resistance of *S*. Typhimurium by modulation of NAD levels via NadR.

### The trifunctional NadR is a conserved c-di-GMP receptor in members of the order Enterobacterales

**Fig 7:**
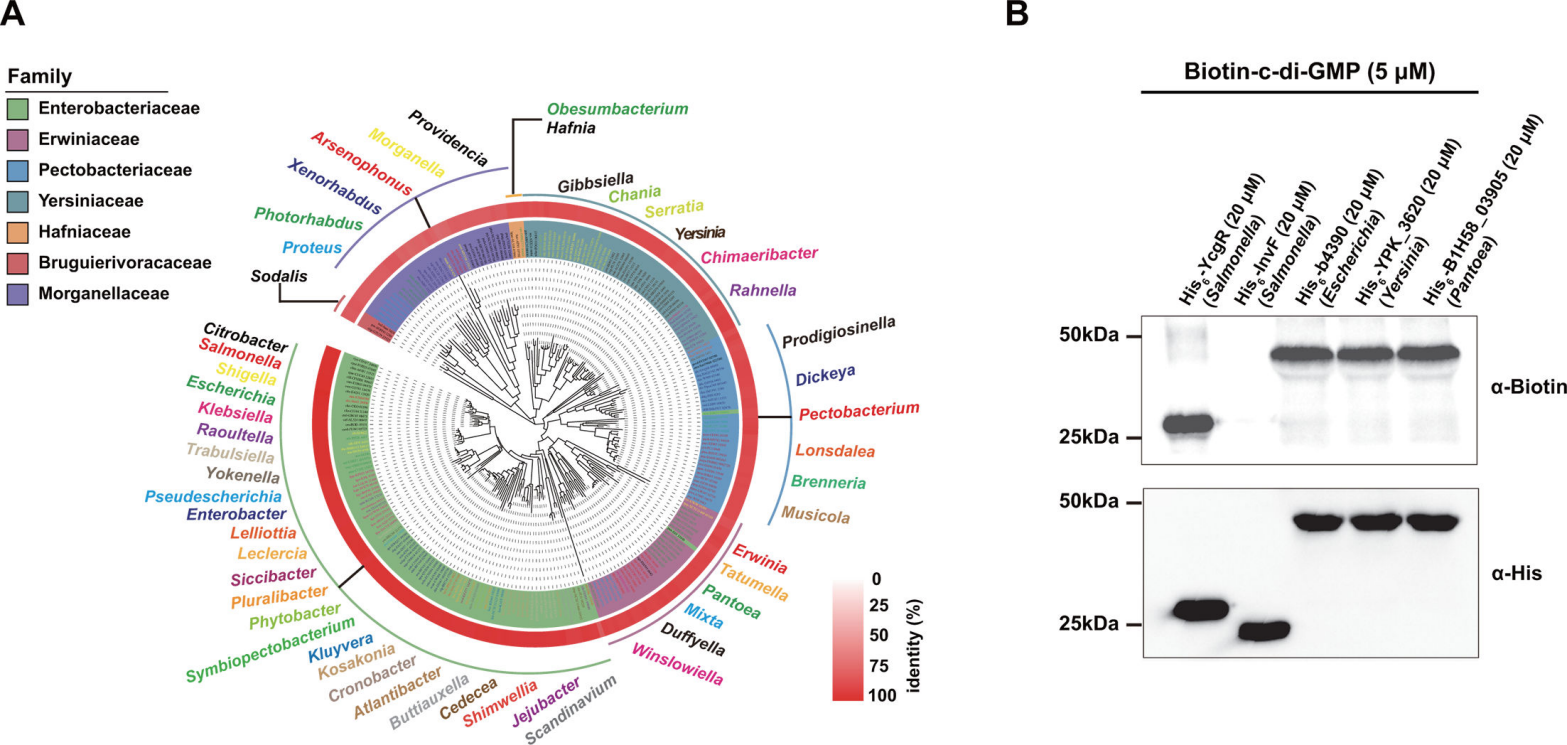
NadR homologs within the order Enterobacterales also bind c-di-GMP. (**A**) Phylogenetic analysis of the NadR family in the order Enterobacterales. NadR homologs were searched by BLASTP against the NCBI non-redundant protein database, and members of the order Enterobacterales were used for phylogenetic analysis (Data S1). The heat map represents the sequence identity between the NadR protein in *S*. Typhimurium and its homologs. (**B**) The UV-crosslinking assay showing the binding of three NadR proteins, His_6_-b4390, His_6_-YPK_3620, and His_6_-B1H58_03905, from *E. coli*, *Y. pseudotuberculosis,* and *P. alhagi*, respectively, to biotinylated c-di-GMP. The blots shown are representative of three independent experiments with similar results.

Bacterial species that harbor the paradigm trifunctional NadR protein originally belonged to the family Enterobacteriaceae (35, 39). However, members of the family Enterobacteriaceae have been reclassified into several families within the order Enterobacterales (29), and thus, the trifunctional NadR is now present in members of the order Enterobacterales. Homologous sequence alignment and molecular phylogenetic analysis indicated that trifunctional NadR, which shares >75% sequence identity with NadR of *S*. Typhimurium, is distributed in families within Enterobacterales (Fig. 7A). To examine whether c-di-GMP binding is a common feature of these NadR proteins, three of them from *E. coli*, *Pantoea alhagi,* and *Yersinia pseudotuberculosis* were expressed and purified, and then their ability to bind c-di-GMP was assessed by the UV-crosslinking assay. As expected, all of them were found to bind c-di-GMP (Fig. 7B). Thus, our results suggest that the trifunctional NadR is a conserved c-di-GMP receptor in members of Enterobacterales, and c-di-GMP-mediated control of NAD synthesis via NadR may represent a common mechanism for the regulation of NAD homeostasis within these bacteria.

## DISCUSSION

c-di-GMP is a highly versatile second messenger that is widely distributed across the bacterial kingdom and has been implicated in the regulation of various important biological processes (10, 32–34, 40–42). Although an array of c-di-GMP-binding effector proteins have been reported, unknown c-di-GMP effectors and their roles in the regulation of cellular functions in response to this signaling molecule still remain to be explored. In the present study, we conducted a c-di-GMP/protein binding assay with biotin-labeled c-di-GMP to screen novel c-di-GMP-binding transcriptional regulators and identified NadR, a transcriptional repressor of NAD synthesis and salvage genes, as a new c-di-GMP receptor in *S*. Typhimurium. Then, we showed that c-di-GMP binds to NadR to inhibit its binding to DNA but stimulate its NMN-AT and RNam-K activities, thus promoting NAD synthesis and enhancing the defense of *S*. Typhimurium against DNA damage. c-di-GMP binding is also detected in the NadR protein from several other species within the order Enterobacterales, suggesting that c-di-GMP-mediated modulation of intracellular NAD homeostasis via NadR is conserved in members of Enterobacterales.

*S*. Typhimurium encodes 17 putative c-di-GMP-metabolizing enzymes and is believed to have a complex c-di-GMP signaling network to cope with the changing environments (4, 43). To date, several c-di-GMP effectors, including the flagellar brake protein YcgR, the cellulose synthesis enzyme BcsA, the T3SS-1 chaperone SicA, the histone-like nucleoid structuring protein H-NS, and the YajQ family protein STM0435, have been identified in *S*. Typhimurium (4, 5, 40, 44, 45). However, while c-di-GMP-responsive transcriptional regulators have been frequently identified in bacterial species such as *Mycobacterium smegmatis* and *Pseudomonas aeruginosa* (10, 32, 46), H-NS is the only identified c-di-GMP-binding transcriptional regulator in *S*. Typhimurium (5). In addition, c-di-GMP was shown to affect the regulatory function of the transcription factor InvF by interaction with its chaperone SicA (4). Here, we identify another transcriptional regulator, NadR, as a direct c-di-GMP effector and thus reveal a previously uncharacterized role of c-di-GMP in the regulation of NAD synthesis. Previous studies have shown that intracellular c-di-GMP levels are controlled in time and space, with various internal and external signals modulating the expression or activity of c-di-GMP-metabolizing enzymes (4, 5, 47–50). In particular, the quorum-sensing signal AI-2, L-arginine, and bile have been reported to influence c-di-GMP metabolism by directly or indirectly targeting DGCs in *S*. Typhimurium (4, 5, 51). It is likely that these signals regulate NAD biosynthesis via modulation of c-di-GMP signaling in *S*. Typhimurium.

The trifunctional NadR protein that originally belonged to the family Enterobacteriaceae and is now distributed within the order Enterobacterales is unusual in that it has three different functional domains (21). In contrast, NadR from bacterial species such as *Haemophilus influenzae* and *Lactococcus lactis* is a bifunctional protein lacking the DNA-binding domain and is thus not a transcriptional repressor (30, 31). Consistent with a previous study (27), our results show that the trifunctional NadR has the ability to bind operator DNA in the absence of ligands (Fig. 3A and B). The transcriptional repressor NadR has been shown to bind two ligands, ATP and NAD, under different physiological conditions (26, 27). In case of low NAD and normal ATP levels, ATP binds to NadR to release it from DNA, allowing transcription of the NAD synthesis and salvage genes (27). When NAD levels are high, NAD binds to the NMN-AT domain of NadR and displaces ATP, restoring the DNA-binding activity of NadR and repressing gene transcription (27). In the present study, we show that c-di-GMP is also a ligand for NadR, and this second messenger plays a similar regulatory role as the inducer ATP, with its binding inhibiting the DNA-binding activity of NadR (Fig. 3C and D). Moreover, when NAD functions as a corepressor by allowing NadR to bind DNA in the presence of ATP, a high level of c-di-GMP abolished the interaction of NadR with DNA (Fig. 4D and E), further indicating that c-di-GMP functions as an inducer of NadR-repressed NAD synthesis and salvage genes. Furthermore, in contrast to NAD, c-di-GMP stimulates the NMN-AT and RNam-K activities of NadR (Fig. 3G and H), suggesting that c-di-GMP plays dual roles as an inducer of NAD biosynthesis *in vivo*. Consequently, an elevation in intracellular c-di-GMP level increased the cytosolic pools of both NAD^+^ and NADH by approximately 1.5-fold, which is comparable to the extent of change in NAD^+^ and NADH concentrations caused by derepression of the transcriptional regulator NrtR via functional impairment of its acetylation site in *M. smegmatis* (25). Thus, our work reveals the implication of c-di-GMP signaling in the NadR-mediated regulation of NAD synthesis and salvage, expanding our understanding of the physiological roles of c-di-GMP in bacteria.

While changes in cellular NAD levels have been widely reported to modulate diverse cellular processes that govern human health and disease (11–13, 38), its role in bacterial physiology is relatively less studied. Some studies have linked cellular NAD levels to bacterial virulence (14, 52–54). Regulation of total NADH and NAD^+^ production by perturbing central metabolism has been shown to affect the virulence of *P. aeruginosa* (52), while mutation of *nrtR* encoding the repressor of the NAD salvage pathway in this bacterium led to loss of fitness and pathogenicity (54). Decreased cytosolic NAD^+^ concentration was shown to be correlated with derepression of the Rex regulon involved in virulence of *Streptococcus agalactiae* (53). Moreover, the increase in NADH levels is suggested to be an evolutionary resistance mechanism of *Chromobacterium violaceum* against antibiotics (14, 55), while depletion of NAD^+^ is a strategy adopted by bacteria to defend against phage infection (14). While the role of NAD^+^ in DNA repair has been well demonstrated (11, 14, 15, 38), our current study shows that c-di-GMP-induced upregulation of total NAD^+^ and NADH pool via targeting NadR enhances the defense of *S*. Typhimurium against DNA damage. In a previous study, c-di-GMP was also shown to positively regulate DNA repair in *Vibrio cholerae* through activation of the DNA repair gene encoding 3-methyladenine glycosylase by the VpsR/VpsT c-di-GMP-dependent cascade (56). c-di-GMP has a similar effect on DNA repair via different signaling pathways in the two bacterial species, suggesting that c-di-GMP may participate in the regulation of DNA repair via various regulatory mechanisms in a wider range of bacteria. Given the highly versatile roles of NAD in energy metabolism, redox, and signal transduction, the effect of c-di-GMP-mediated modulation of NAD levels on the biology of *Salmonella* needs to be further explored.

In summary, this study identifies NadR as a c-di-GMP effector and provides mechanistic insights into how NadR-mediated transcriptional repression of genes involved in NAD *de novo* synthesis and salvage might be counteracted by this second messenger. Our study establishes for the first time a direct link between c-di-GMP signaling and bacterial NAD synthesis. Since cellular c-di-GMP levels are modulated in response to various internal and external cues, our work paves the way for further exploration of environmental and physiological conditions that regulate NAD synthesis and salvage in bacteria via c-di-GMP signaling.

## MATERIALS AND METHODS

### Bacterial strains and growth conditions

All strains and plasmids used in this study are listed in Table S2. All primers used in this study were designed using Primer Premier 5.0 (Premier Biosoft), and their sequences are listed in Table S3. *S*. Typhimurium strains and *E. coli* strains were grown in LB medium under aerobic conditions at 37°C unless specified otherwise. If relevant, antibiotics were used at the following concentrations: 50  µg mL^−1^ kanamycin, 20  µg mL^−1^ streptomycin, or 20  µg mL^−1^ chloramphenicol. The mutants of *S*. Typhimurium were constructed using the CRISPR-Cas9 system (57). For complementation or overexpression, derivatives of PKT100 were transformed into relevant strains.

### Cloning, expression, and purification of recombinant proteins

The target DNA fragment was amplified by PCR using *S*. Typhimurium wild-type single colony or genomic DNA as a template. The linearized vector pET28a was ligated with the target gene segment using OK Clone DNA Ligation Kit (Accurate Biology, Hunan, China), and the resultant product was thermally transformed into *E. coli* TG1 competent cells. Then, the recombinant plasmid was heat-shock transformed into competent *E. coli* BL21 (DE3) cells. *E. coli* BL21(DE3) strains carrying the pET-28a derivatives were cultivated at 37°C until an OD_600_ of 0.4, and then IPTG was added at a final concentration of 0.25 mM. After induction with IPTG at low temperature (16°C) for 10 h, bacterial cells were harvested by centrifugation at 4°C and 8,000 rpm for 3 min, followed by removal of the supernatant. Bacterial cell pellets were resuspended, disrupted by sonication, and then recombinant proteins were purified with Ni^2+^-NTA His-bind resin (Novagen, Madison, WI, USA) according to the manufacturer’s instructions. The purified proteins were analyzed by SDS-PAGE and divided into small portions for storage at −80°C. All recombinant proteins used in this study contained an N-terminal His_6_ tag.

### UV-crosslinking assays

His_6_-tagged proteins (20 µM) were incubated with biotinylated c-di-GMP (5 µM; Biolog, Germany) in 5× Tris buffer with gentle mixing at room temperature for 20 min. When needed, unlabeled nucleotides or NAD were added to the reaction mixture simultaneously with biotinylated c-di-GMP. The sample was then exposed to UV light for 30 min, followed by the addition of 5× SDS loading buffer and denaturation at 100°C for 10 min. The denatured proteins were subsequently separated by SDS-PAGE. The resolved proteins were transferred onto a nylon membrane (Millipore), followed by soaking in 0.5× TBE buffer under UV light for 10 min. The membrane was then blocked with Blocking buffer (Thermo Scientific) for 1 h at room temperature, followed by incubation with horseradish peroxidase-labeled streptavidin (Thermo Scientific, cat# 21126) at a 1:5,000 dilution for 15 min. After incubation, the membrane was washed six times with Wash buffer (Thermo Scientific) at room temperature. The chemiluminescence signal was subsequently detected using an enhanced chemiluminescence (ECL) reagent (GE Healthcare). For control, a membrane exposed to UV irradiation was blocked and incubated with a 1:5,000 dilution of mouse anti-His (Abways, Shanghai, China, cat# AB0002), followed by incubation with a 1:10,000 dilution of goat anti-mouse secondary antibodies (DIYIBIO, China, cat# DY60203) before detecting signals using the ECL system.

### ITC analysis

ITC assays were performed at 25°C using a Nano-ITC Low Volume isothermal calorimeter (TA Instruments, New Castle, DE, USA) controlled by ITC Run software. All proteins were dialyzed against a Tris buffer (25 mM Tris and 300 mM NaCl, pH 7.5) and then diluted to 5 µM, while the ligands c-di-GMP, c-di-AMP, cGMP, ATP, and NAD were diluted to 150 µM with the same dialysis buffer. All samples were degassed before titrations. Proteins are added to the sample pool, and ligands are added to the titration needle. There were 25 injections for each sample, and the stirring rate was 200 rpm. In control experiments, ligand solutions were titrated into the buffer in the sample pool to obtain the heats of dilution. The NanoAnalyze software provided by the manufacturer was used to analyze the ITC data, and the binding curve was fit to an independent single-binding site model after subtracting the heat of ligand dilution (58).

### Molecular docking analysis

The 3D structure of NadR in *S*. Typhimurium was downloaded from the AlphaFold Protein Structure Database (AFDB accession number: AF-P24518-F1; https://alphafold.ebi.ac.uk/) (59). The 3D structures of c-di-GMP, ATP, and NAD were extracted from the crystal structures of the protein-ligand complexes 2L74 (https://www.rcsb.org/structure/2L74), 5IT5 (https://www.rcsb.org/structure/5IT5), and 3CGD (https://www.rcsb.org/structure/3CGD), respectively. AutoDock4 was used to add hydrogen and compute Gasteiger charges for NadR and the three ligands (60). Flexible torsions of c-di-GMP, ATP, and NAD were assigned using Autodock4 (60). Docking simulation was performed by AutoDock Vina 1.1.2 (61), with the best ligand-binding mode selected based on the lowest docking energy. The three-dimensional docking conformations were displayed with PyMOL (http://www.pymol.org), and protein-ligand interactions were analyzed using LigPlot^+^ (37).

### EMSAs

For EMSAs, 50 ng of DNA probes were incubated with NadR or its variants in a Tris buffer (20 mM Tris, 100 mM NaCl, 10% glycerol, 4 mM MgCl_2_, 1% NP-40, and 1 mM dithiothreitol, pH 7.5) in a 25 µL reaction system for 20 min at room temperature. When needed, nucleotides (c-di-GMP, cGMP, or c-di-AMP), ATP, and/or NAD were added simultaneously with the protein to the reaction system. After incubation, the samples were subjected to 6% native polyacrylamide gels and run in 0.5× Tris-borate-EDTA buffer at 100 V for 2 h. Then, the gels were stained with SYBR Safe DNA gel stain (Invitrogen) for 10 min, followed by detection and imaging of DNA probes by a fluorescent imaging system (Tanon 5200Multi, China).

### *In vitro* NMN-AT and RNam-K activity assays for NadR

*In vitro* NMN-AT and RNam-K activity assays were performed as described previously (27). In brief, the reactions were run at 37°C in a 0.2 mL reaction mixture containing 1 mM RNam (for RNam-K assays) or 3 mM NMN (for NMN-AT assays), 5 mM MgCl_2_, 1 mM ATP, 20 µM NadR, and 100 mM Tris-HCl (pH 7.6) with and without 200 µM c-di-GMP. The reaction was initiated by the addition of ATP and stopped by heating (98°C for 90 s). The denatured protein was removed through centrifugation, and the supernatant was filtered through a 0.22 µm membrane. Then, samples were subjected to HPLC analysis with a C18 reversed-phase column and a UV detector. The mobile phase consisted of 95% A (10 mM sodium acetate in water acidified with acetic acid to pH 4.5) and 5% B (methanol). The detection wavelength was 254 nm. The products NAD^+^ and NMN in the NMN-AT and RNam-K activity assays, respectively, were quantified from the standard curves obtained using known concentrations of NAD^+^ and NMN.

### c-di-GMP quantification

The cellular c-di-GMP quantification was performed as described previously (5). Relevant strains of *S*. Typhimurium were cultivated in LB medium until an OD_600_ of 2.0. 15 mL of culture was centrifuged, and then the cell pellets were washed twice with PBS, followed by resuspension in 500 µL of extraction buffer (acetonitrile/methanol/water, 40:40:20, vol/vol/vol) and incubation at 4°C for 15 min. After incubating in a 95°C water bath for 10 min and cooling in an ice bath for 15 min, the suspension was centrifuged at 10,000 rpm at 4°C for 10 min, and the supernatant was transferred to a new EP tube. The remaining bacterial pellets were treated with 500 µL of extraction buffer on ice again for 15 min and centrifuged to obtain the supernatant. This extraction step was repeated twice, and the supernatants from all three extractions were collected together. The pooled supernatants were freeze-dried to remove all solvents, and then the dried product was dissolved in 200 µL of distilled water. The samples were analyzed by liquid chromatography-tandem mass spectrometry. Levels of c-di-GMP were normalized to the number of bacterial cells (10^7^ cfu) for each sample.

### β-galactosidase activity assays

The construction of the fusion reporter strains is performed following a previous method (4). The promoter-lacZ fusion reporter strains were grown in LB medium until an OD_600_ of 2.0. A total of 50 µL of bacterial culture was added to 450 µL of Z-buffer (100 mM Na_2_HPO_4_, 10 mM KCl, 40 mM NaH_2_PO_4_, 1 mM MgSO_4_, and 5.4 µL mL^−1^ β-mercaptoethanol), 20 µL chloroform, 10 µL of 0.1% SDS and incubated at 30°C for 1 h. Then, 100 µL substrate solution (60 mM Na_2_HPO_4_, 40 mM NaH_2_PO_4_, 2.7 µL mL^−1^ β-mercaptoethanol, and 4 mg mL^−1^ ONPG) was added for β-galactosidase activity detection, and 250 µL of 1 M Na_2_CO_3_ solution was used to terminate the reaction. The OD_420_ and OD_550_ values of the reaction solution were measured with a microplate reader. The β-galactosidase activity was calculated and expressed as Miller units (62).

### RNA extraction and qRT-PCR assays

The *S*. Typhimurium-related strains were grown in LB medium until an OD_600_ of 2.0. A total of 500 µL of bacterial culture was harvested, and then total RNA was extracted using the MolPure Bacterial RNA Kit (Yeasen Biotechnology, Shanghai, China). Five hundred nanograms of total RNA was reverse transcribed to cDNA by using TransScript II One-Step gDNA Removal and cDNA Synthesis SuperMix (TransGen Biotech, Beijing, China). qPCR was then performed using KAPA SYBR FAST qPCR Kit (Kapa Biosystems, USA) in a LightCycler 96 thermocycler (Roche), with the 16S rRNA used as an internal control.

### ChIP-qPCR assays

*S*. Typhimurium strain expressing *in situ*-tagged HA-NadR and HA-NadR_E179A_ was generated by using the CRISPR-Cas9 system (57). *S*. Typhimurium strains were grown in LB medium until an OD_600_ of 2.0, and 2 mL of bacterial cells were harvested. The ChIP assay was performed as described in a previous study (5). Briefly, cultures were fixed with 1% formaldehyde for 20 min at room temperature to obtain stable DNA-protein complexes, and the crosslinking reaction was quenched with 125 mM glycine for 5 min. Cells were harvested and washed with PBS (pH 7.0) three times and then resuspended in 1 mL lysis buffer (25 mM Tris, 150 mM NaCl, 1 mM EDTA, 0.1% Triton X-100, and 0.1% SDS, pH 7.5), followed by sonication to generate DNA fragments of 100–500 bp. The sonicated chromatin was incubated with either no antibody (mock-IP) or anti-HA (1:2,000 dilution, Abways, Shanghai, China) for 10 h at 4°C, followed by enrichment with protein A magnetic beads (Beyotime, Shanghai, China) that had been washed with PBS (pH 7.0). The immunoprecipitated complexes were eluted three times with the elution buffer (50 mM Tris-HCl, pH 7.5, 10 mM EDTA, and 1% SDS) at 65°C for 15 min. Crosslinks were then reversed by incubation at 65°C for 6 h in 0.5× elution buffer supplemented with 250 µg mL^−1^ proteinase K. The DNA fragments were isolated using a phenol/chloroform/isoamyl alcohol extraction solution (25:24:1), and the relative enrichment of each fragment was determined by qPCR. The ChIP-qPCR signals were normalized to their respective DNA inputs. Background signals from mock samples without the addition of the anti-HA antibody were subtracted in the final analysis.

### Determination of intracellular NAD^+^ and NADH concentrations

All strains were grown in LB medium until an OD_600_ of 2.0. Two milliliters of bacterial cultures was harvested and washed twice with ddH_2_O at 4°C. The intracellular level of both NAD^+^ and NADH was determined using an NAD^+^/NADH kit (Sino Best Biological Technology) according to the manufacturer’s instructions (25). Levels of NAD^+^/NADH were normalized to the number of bacterial cells (10^8^ cfu) for each sample.

### UV stress assays

The UV stress assays were performed as described previously with some modifications (63). *S*. Typhimurium strains grown overnight in LB liquid medium at 37°C were subcultured at a 1:100 dilution in fresh LB medium until an OD_600_ of 2.0. The cultures were exposed to 150 mJ cm^−2^ of UV light (254 nm) at a dose rate of 0.5 mJ cm^−2^ s^−1^. The irradiated cultures were then serially diluted and spread on LB agar plates to determine the number of viable cells. The survival rate was calculated as the ratio of viable cells upon UV irradiation to viable cells without UV irradiation.

### Phylogenetic analyses

NadR homologs were searched by BLASTP against the NCBI non-redundant protein database. Multiple sequence alignment was performed using MUSCLE implemented in MEGA 11 to align the retrieved NadR homologs. Phylogenetic trees were constructed using the neighbor-joining method in MEGA 11 (https://www.megasoftware.net/dload_win_gui) (64), with 1,000 bootstrap replicates to assess the reliability of the inferred relationships. The Interactive Tree of Life (https://itol.embl.de/) (65) was used to adjust the topological structure of the phylogenetic tree, add annotation information, and beautify the phylogenetic tree.

### Statistical analysis

Statistical analyses of all data were performed using Microsoft Excel 2019 or GraphPad Prism 8.0 (GraphPad, USA). All experimental data were expressed as the means ± standard deviation, and a *P*-value less than 0.05 was considered statistically significant. All graphs were created using Adobe Illustrator 2020 (CS6; Adobe, Mountain View, CA, USA).

## SUPPLEMENTAL MATERIAL

Data S1 mbio.01982-25-s0001.xlsx NadR homologs within the order Enterobacterales.

Supplemental Material mbio.01982-25-s0002.pdf Supplemental figures and tables.

ASM does not own the copyrights to Supplemental Material that may be linked to, or accessed through, an article. The authors have granted ASM a non-exclusive, world-wide license to publish the Supplemental Material files. Please contact the corresponding author directly for reuse.
