# Supplementary material for: c-di-GMP regulates bacterial NAD biosynthesis via targeting the transcriptional repressor NadR
Source: mBio. 2025 Aug 18;16(9):e01982-25. doi: 10.1128/mbio.01982-25 (PMC12421817; doi:10.1128/mbio.01982-25)
Supplement: Supplemental Material — Supplemental figures and tables. [file mbio.01982-25-s0002.pdf]

Supplementary Information

c-di-GMP regulates bacterial NAD biosynthesis via targeting the transcriptional repressor NadR

Lianying Mao†, Jialin Li†, Xinyi Huo, Wenguang Yang, Heng Zhang, Chongyi Duan, Xihui Shen, Lei Zhang*

State Key Laboratory for Crop Stress Resistance and High-Efficiency Production, Shaanxi Key Laboratory of Agricultural and Environmental Microbiology, College of Life Sciences, Northwest A&F University, Yangling, Shaanxi 712100, China.

†These authors contributed equally to this work.

***For correspondence:**

Lei Zhang (zhanglei0075@nwsuaf.edu.cn).

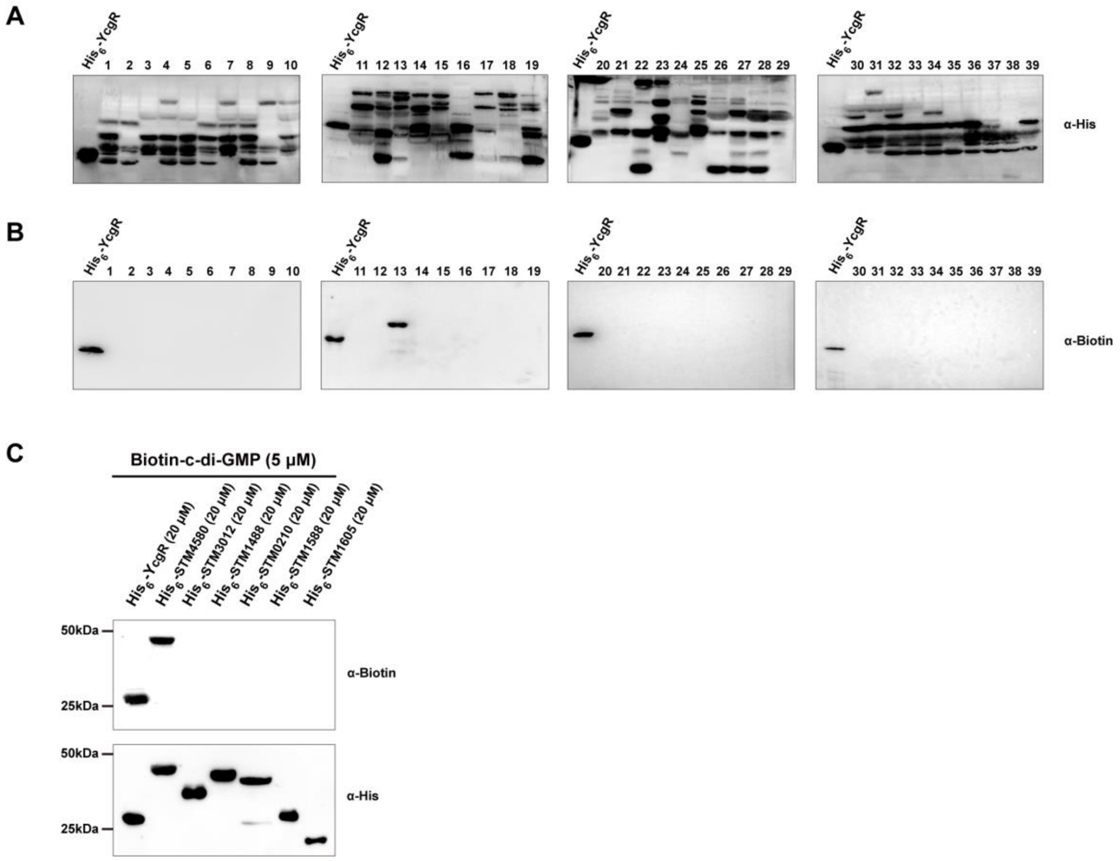

Fig. S1 Identification of potential c-di-GMP receptors from the *S*. Typhimurium transcription factor library. **A** The UV-crosslinking assays detecting the co-purification of 39 groups of mixed recombinant transcriptional regulators. The co-purified samples were separated by SDS-PAGE and probed with the anti-His antibody. **B** The UV-crosslinking assays detecting the binding of the 39 groups of His$_6$-tagged mixed recombinant transcriptional regulators to biotinylated c-di-GMP. The reaction samples were separated by SDS-PAGE and probed with the anti-biotin antibody streptavidin HRP. **C** The UV-crosslinking assay detecting the binding of the six separately purified proteins in group 13 to biotinylated c-di-GMP. **A-C** His$_6$-YcgR was used as a positive control. Blots shown are representative of three independent experiments with similar results.

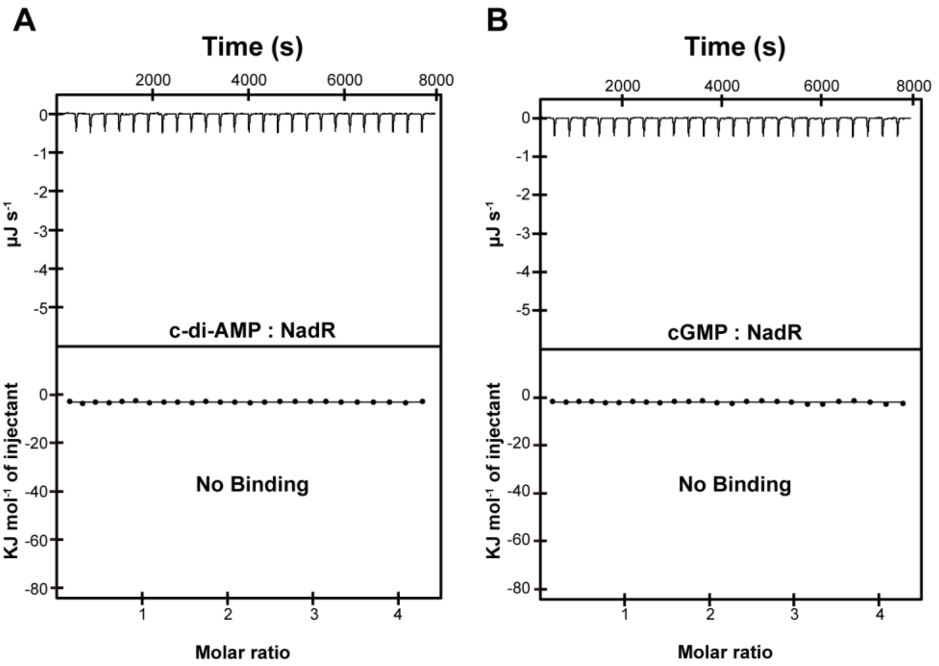

Fig. S2 ITC detecting the binding of the NadR protein of *S.* Typhimurium with c-di-AMP (**A**) and cGMP (**B**). The original titration data and integrated heat measurements are shown in the upper and lower plots, respectively. The heats of ligand dilution were subtracted and the corrected data were fit to a one-site binding model. Isotherms shown are one representative of three independent experiments with similar results.

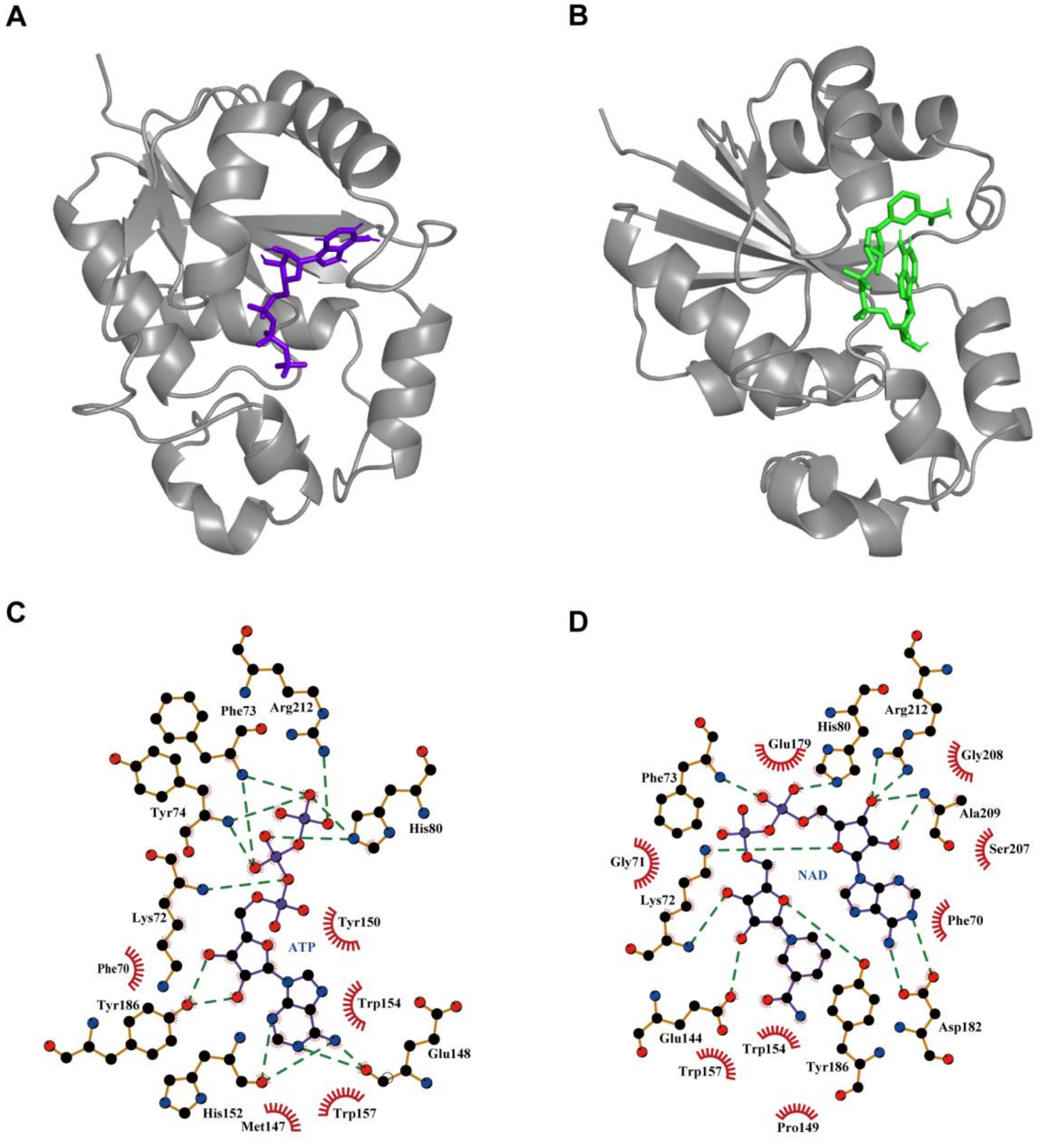

Fig. S3 Predicted binding mode of ATP or NAD to the NMN-AT domain of NadR. **A, B** Predicted 3D model of NadR-ATP (**A**) and NadR-NAD (**B**) complexes. **C, D** Schematic of the predicted contacts of ATP (**C**) or NAD (**D**) with NadR from the binding conformations. Potential hydrogen bonds are indicated as green dashed lines.

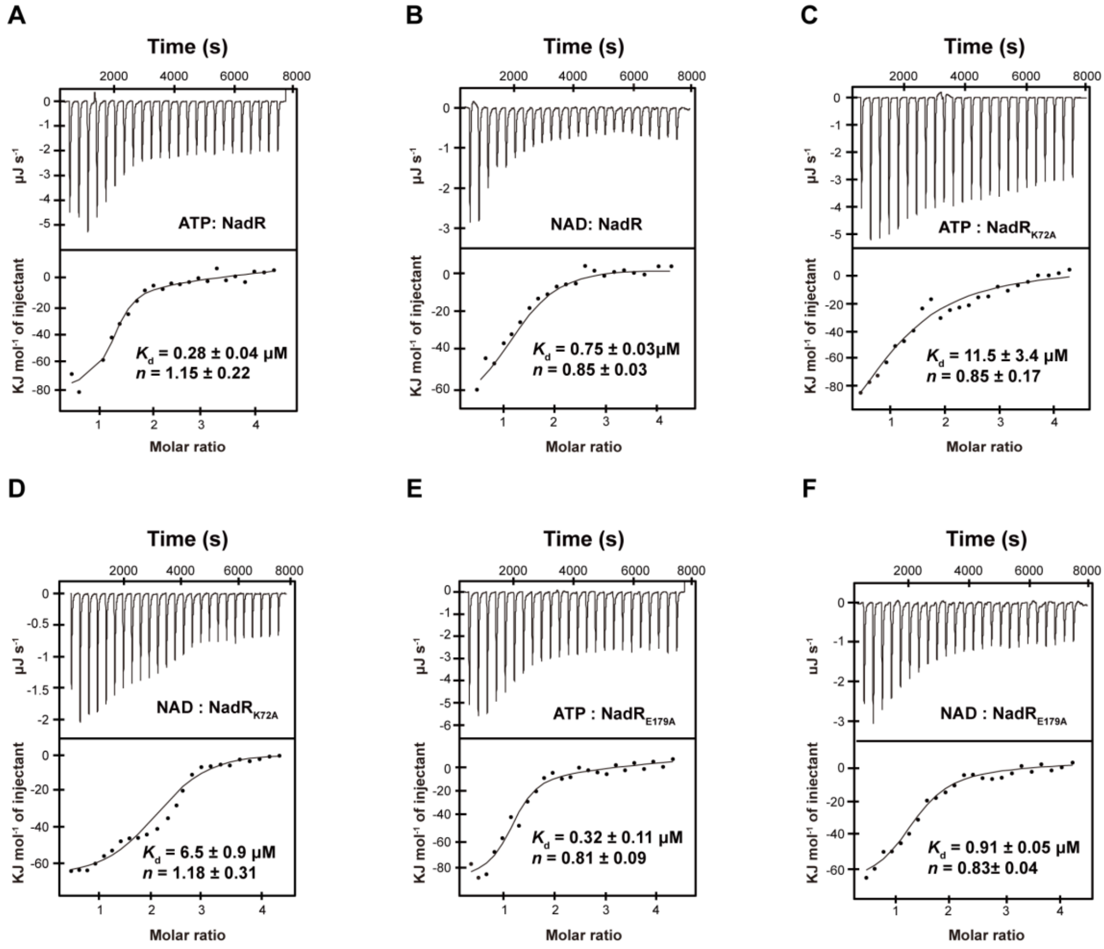

Fig. S4. ITC assays for the interactions of wild-type NadR and its variants K72A or E179A with ATP and NAD. **A**, **B** NadR interacts with ATP (**A**) and NAD (**B**) with high affinity. **C**, **D** ITC assays for the interactions of the NadR variant K72A with ATP (**C**) and NAD (**D**). **E**, **F** ITC assays for the interactions of the NadR variant E179A with ATP (**E**) and NAD (**F**). **A**-**F** Isotherms shown are one representative of three independent experiments with similar results. The K_d and *n* values are presented as mean ± s.d. of the three independent experiments.

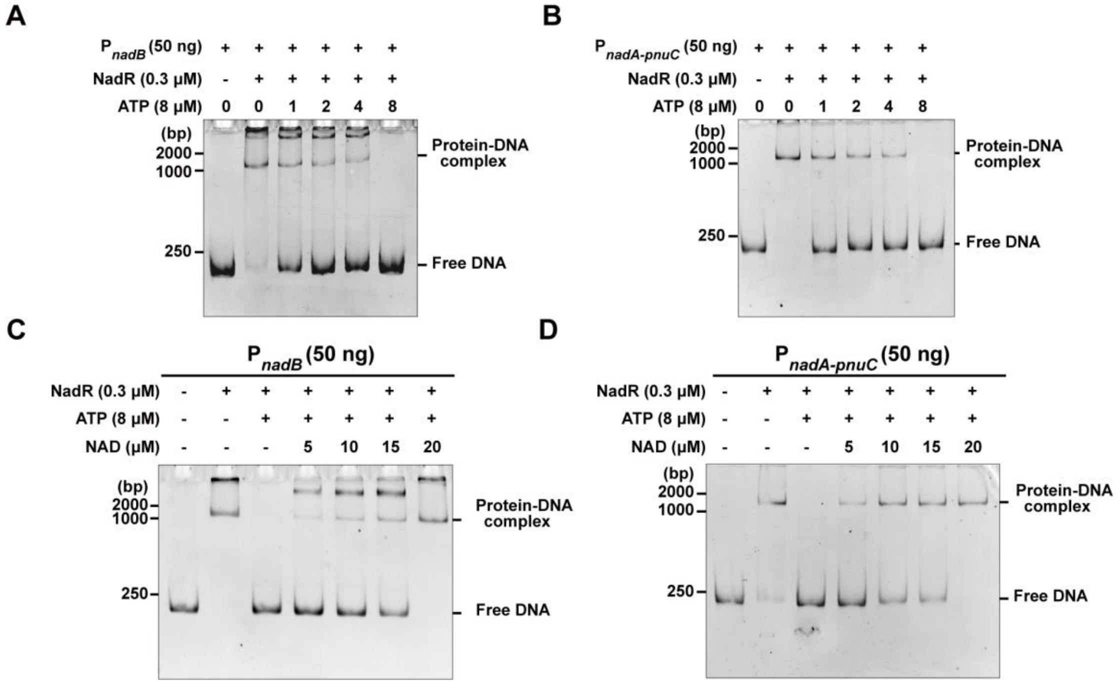

Fig. S5 EMSAs for binding of NadR to promoters of the *nadB* gene and the *nadA*-*pnuC* operon in the absence or presence of ATP and/or NAD. **A, B** ATP inhibits the binding of NadR to the promoters of *nadB* (**A**) and *nadA*-*pnuC* (**B**). **C, D** NAD allows NadR to bind the promoters of *nadB* (**C**) and *nadA*-*pnuC* (**D**) in the presence of ATP. **A-D** Gels shown are representative of three independent experiments with similar results.

Supplementary Tables

Table S1. The 163 successfully expressed His$_6$-tagged transcription factors from *S.* Typhimurium

NCBI gene ID	KEGG ID	Functional annotation
WP_000093829.1	STM4580	multifunctional transcriptional regulator/nicotinamide-nucleotide adenylyltransferase/ribosylnicotinamide kinase NadR
WP_000002497.1	STM0606	LysR family transcriptional regulator
WP_000010814.1	STM2839	nitric oxide reductase transcriptional regulator YgaA
WP_000021514.1	STM2759	SgrR family transcriptional regulator
WP_000024658.1	STM2180	LysR family transcriptional regulator
WP_000026949.1	STM2345	LacI family DNA-binding transcriptional regulator
WP_000033840.1	STM4417	myo-inositol utilization transcriptional regulator IolR
WP_000044409.1	STM2982	glycine cleavage system transcriptional regulator GcvA
WP_000059693.1	STM4025	DeoR/GlpR transcriptional regulator
WP_000081340.1	STM3667	IclR family transcriptional regulator YiaJ
WP_000081378.1	STM0344	transcriptional regulator
WP_000083126.1	STM1122	TetR/AcrR family transcriptional regulator
WP_000091471.1	STM3452	transcriptional regulator YheO
WP_000106992.1	STM4297	transcriptional regulator MelR
WP_000121043.1	STM1575	TetR family transcriptional regulator
WP_000126152.1	STM2794	GntR family transcriptional repressor
WP_000131699.1	STM0693	ferric iron uptake transcriptional regulator
WP_000133618.1	STM4381	HTH-type transcriptional regulator UlaR
WP_000134791.1	STM3025	transcriptional regulator
WP_000174317.1	STM3830	D-galactonate utilization transcriptional regulator DgoR
WP_000176719.1	STM2281	LysR family transcriptional regulator
WP_000193201.1	STM3687	MltR family transcriptional regulator
WP_000198318.1	STM0410	YafY family transcriptional regulator
WP_000201030.1	STM3011	HTH-type transcriptional regulator GalR
WP_000201080.1	STM1555	LacI family DNA-binding transcriptional regulator
WP_000213163.1	STM3860	SgrR family transcriptional regulator
WP_000215563.1	STM1510	GntR family transcriptional regulator
WP_000216297.1	STM2912	LysR family transcriptional regulator
WP_000225097.1	STM1488	ROK family transcriptional regulator
WP_000234826.1	STM1805	fatty acid metabolism transcriptional

		regulator FadR
WP_000242758.1	STM3466	cAMP-activated global transcriptional regulator CRP
WP_000243999.1	STM2867	Transcriptional regulator SirC
WP_000269523.1	STM1429	LysR family transcriptional regulator
WP_000274596.1	STM4117	helix-turn-helix transcriptional regulator
WP_000282119.1	STM0764	LysR family transcriptional regulator
WP_000284836.1	STM1355	AraC family transcriptional regulator
WP_000332219.1	STM4423	myo-inositol utilization transcriptional regulator ReiD
WP_000339633.1	STM0563	AraC family transcriptional regulator
WP_000354963.1	STM4270	LysR family transcriptional regulator
WP_000358042.1	STM3607	LysR family transcriptional regulator
WP_000359201.1	STM1677	LysR family transcriptional regulator
WP_000366538.1	STM1523	LysR family transcriptional regulator
WP_000382926.1	STM3340	transcriptional regulator NanR
WP_000383509.1	STM4511	hypochlorite stress DNA-binding transcriptional regulator HypT
WP_000414259.1	STM1625	LysR family transcriptional regulator
WP_000423170.1	STM3121	LysR family transcriptional regulator
WP_000423362.1	STM0692	tricarballylate utilization LysR family transcriptional regulator TcuR
WP_000432699.1	STM2875	transcriptional regulator HilD
WP_000433018.1	STM3876	transcriptional regulator AsnC
WP_000433674.1	STM1127	MurR/RpiR family transcriptional regulator
WP_000434062.1	STM2145	GntR family transcriptional regulator
WP_000438890.1	STM2420	LysR family transcriptional regulator
WP_000448638.1	STM3794	DeoR family transcriptional regulator
WP_000533542.1	STM0835	manganese-binding transcriptional regulator MntR
WP_000534902.1	STM0014	LysR family transcriptional regulator
WP_000543533.1	STM0415	transcriptional regulator NrdR
WP_000545327.1	STM0859	LysR family transcriptional regulator
WP_000548308.1	STM2201	LysR family transcriptional regulator
WP_000565119.1	STM2436	transcriptional regulator PtsJ
WP_000569786.1	STM3964	HTH-type transcriptional regulator MetR
WP_000572568.1	STM3602	GntR family transcriptional regulator
WP_000579793.1	STM1279	helix-turn-helix transcriptional regulator
WP_000597928.1	STM0952	LysR family transcriptional regulator
WP_000605987.1	STM1924	flagellar transcriptional regulator FlhC
WP_000606288.1	STM2330	transcriptional regulator LrhA
WP_000611914.1	STM1660	fumarate/nitrate reduction transcriptional regulator Fnr

WP_000613185.1	STM2919	DeoR/GlpR transcriptional regulator
WP_000628631.1	STM2191	HTH-type transcriptional regulator GalS
WP_000633676.1	STM3790	transcriptional regulator UhpA
WP_000636696.1	STM3693	transcriptional regulator LldR
WP_000648534.1	STM0256	LysR family transcriptional regulator
WP_000661875.1	STM0869	TetR/AcrR family transcriptional regulator
WP_000667440.1	STM3547	DeoR family transcriptional regulator
WP_000678254.1	STM0164	DeoR/GlpR transcriptional regulator
WP_000723776.1	STM3358	GntR family transcriptional regulator
WP_000724553.1	STM3533	IclR family transcriptional regulator
WP_000738617.1	STM0020	winged helix-turn-helix transcriptional regulator
WP_000741798.1	STM3014	LysR family transcriptional regulator
WP_000749366.1	STM3848	HTH-type transcriptional regulator YidZ
WP_000749979.1	STM2749	transcriptional regulator
WP_000753665.1	STM2454	HTH-type transcriptional regulator EutR
WP_000764553.1	STM1444	transcriptional regulator SlyA
WP_000776233.1	STM1713	HTH-type transcriptional regulator CysB
WP_000801273.1	STM0549	fimbria biosynthesis transcriptional regulator FimZ
WP_000826514.1	STM2414	putative DNA-binding transcriptional regulator
WP_000828345.1	STM3064	DNA-binding transcriptional regulator ArgP
WP_000836268.1	STM0430	phosphonate utilization transcriptional regulator PhnR
WP_000841337.1	STM4094	DNA-binding transcriptional regulator CytR
WP_000841654.1	STM4507	Uxu operon transcriptional regulator
WP_000852387.1	STM0104	arabinose operon transcriptional regulator AraC
WP_000852810.1	STM4099	met regulon transcriptional regulator MetJ
WP_000854956.1	STM3785	GntR family transcriptional regulator
WP_000859023.1	STM0333	LysR family transcriptional regulator
WP_000876817.1	STM2195	helix-turn-helix transcriptional regulator
WP_000891805.1	STM3678	helix-turn-helix transcriptional regulator
WP_000894170.1	STM2292	IclR family transcriptional regulator
WP_000907242.1	STM1899	YebC/PmpR family DNA-binding transcriptional regulator
WP_000921674.1	STM4315	AraC family transcriptional regulator
WP_000929420.1	STM0210	DNA-binding transcriptional regulator CdaR
WP_000944156.1	STM0552	fimbria biosynthesis transcriptional regulator FimW

WP_000944714.1	STM2275	GntR family transcriptional regulator
WP_000968354.1	STM3012	LacI family DNA-binding transcriptional regulator
WP_000973433.1	STM0446	transcriptional regulator BolA
WP_000975956.1	STM2265	bifunctional DNA-binding transcriptional regulator/O6-methylguanine-DNA methyltransferase Ada
WP_000983393.1	STM1315	transcriptional regulator ChbR
WP_000983441.1	STM3215	PadR family transcriptional regulator yqjI
WP_000995703.1	STM2572	MurR/RpiR family transcriptional regulator
WP_000998789.1	STM3175	AraC family transcriptional regulator
WP_001020583.1	STM0354	Au(I) sensor transcriptional regulator GolS
WP_001022463.1	STM2575	LysR family transcriptional regulator
WP_001025919.1	STM4125	DNA-binding transcriptional regulator OxyR
WP_001041823.1	STM1437	TetR/AcrR family transcriptional regulator
WP_001050870.1	STM3736	LysR family transcriptional regulator
WP_001083896.1	STM3262	DeoR/GlpR transcriptional regulator
WP_001088593.1	STM1704	DeoR/GlpR transcriptional regulator
WP_001094910.1	STM3245	transcriptional regulator TdcA
WP_001109831.1	STM2424	LysR family transcriptional regulator
WP_001115420.1	STM0115	transcriptional regulator LeuO
WP_001119592.1	STM1588	colanic acid/biofilm transcriptional regulator yncC
WP_001139040.1	STM0109	HTH-type transcriptional regulator SgrR
WP_001171579.1	STM3357	GntR family transcriptional regulator
WP_001181297.1	STM4455	HTH-type transcriptional regulator TreR
WP_001188509.1	STM4322	transcriptional regulator
WP_001190057.1	STM3584	nickel-responsive transcriptional regulator NikR
WP_001201297.1	STM4402	winged helix-turn-helix transcriptional regulator
WP_001217074.1	STM4295	AraC family transcriptional regulator
WP_001232871.1	STM3522	sigma 54-dependent transcriptional regulator
WP_001235493.1	STM1683	transcriptional regulator TyrR
WP_001237934.1	STM2785	transcriptional regulator TctD
WP_001238163.1	STM0456	SgrR family transcriptional regulator
WP_001240418.1	STM2160	HTH-type transcriptional regulator MlrA
WP_001241346.1	STM2544	Fe-S cluster assembly transcriptional regulator IscR
WP_001244065.1	STM4481	LacI family DNA-binding transcriptional

		regulator
WP_001246659.1	STM2866	transcriptional regulator
WP_001247395.1	STM0030	LysR family transcriptional regulator
WP_001250362.1	STM0029	transcriptional regulator
WP_001262195.1	STM1842	DNA-binding transcriptional regulator KdgR
WP_001274847.1	STM3778	DNA-binding transcriptional regulator
WP_001275362.1	STM3124	LuxR family transcriptional regulator
WP_001278062.1	STM3759	transcriptional regulator
WP_001282817.1	STM0634	DNA-binding transcriptional regulator
WP_001283048.1	STM4073	transcriptional regulator LsrR
WP_001293638.1	STM1547	winged helix-turn-helix transcriptional regulator
WP_001518759.1	STM1541	FadR family transcriptional regulator
WP_001519915.1	STM4068	GntR family transcriptional regulator
WP_001526875.1	STM2644	LysR family transcriptional regulator
WP_001541181.1	STM3897	HTH-type transcriptional regulator HdfR
WP_001541637.1	STM3800	DNA-binding transcriptional regulator DsdC
WP_001541751.1	STM3163	AraC family transcriptional regulator
WP_001576357.1	STM3084	FadR family transcriptional regulator
WP_001670738.1	STM1618	DeoR/GlpR transcriptional regulator
WP_001738208.1	STM1265	helix-turn-helix transcriptional regulator
WP_010989081.1	STM3696	LacI family DNA-binding transcriptional regulator
WP_010989088.1	STM4042A	helix-turn-helix transcriptional regulator
WP_000013294.1	STM4049	HTH-type transcriptional activator RhaR
WP_000365798.1	STM3908	HTH-type transcriptional activator IlvY
WP_000371685.1	STM4586	MDR efflux pump AcrAB transcriptional activator RobA
WP_000412686.1	STM4266	redox-sensitive transcriptional activator SoxR
WP_000416272.1	STM4241	zinc uptake transcriptional repressor Zur
WP_000431550.1	STM4548	DNA-binding transcriptional activator BglJ
WP_000450103.1	STM0864	DNA-binding transcriptional repressor DeoR
WP_000730239.1	STM3543	gluconate operon transcriptional repressor GntR
WP_001738297.1	STM1691	phage shock protein operon transcriptional activator

Table S2. Bacterial strains and plasmids used in this study

Strains and plasmids	Relevant characteristics*	Source
**Strains**		
E.coli		
TG1	Host for cloning	Laboratory stock
BL21(DE3)	Host for expression vector pET-28a	Laboratory stock
S17-1λpir	λ-pir lysogen of S17-1, F *thi pro hsdR* [RP4-2 Tc::Mu Km::Tn7(Tp Sm)]	Laboratory stock
***S*.typhimurium**		
SL1344	Wild-type	Laboratory stock
Δ*nadR*	*nadR* deletion mutant in SL1344	This study
nadR(E179A)	SL1344 Wild-type strain witn the E179A mutation of *nadR*	This study
Δ*nadR*(*nadR*)	*nadR* deletion mutant containing pKT100-*nadR* vector	This study
SL1344(*STM0385*)	SL1344 Wild-type strain over-expressing *STM0385*	This study
SL1344(*STM3611*)	SL1344 Wild-type strain over-expressing *STM3611*	This study
Δ*nadR(STM0385*)	*nadR* deletion mutant over-expressing *STM0385*	This study
Δ*nadR*(*STM3611*)	*nadR* deletion mutant over-expressing *STM3611*	This study
nadR(E179A)(*STM0385*)	*nadR*(E179A) deletion mutant over-expressing *STM0385*	This study
nadR(E179A)(*STM3611*)	*nadR*(E179A) deletion mutant over-expressing *STM3611*	This study
SL1344(pDM4-*nadA-pnuC* CDA::lacZ)	SL1344 Wild-type strain containing pKT100 vector and pDM4-*nadA-pnuC* CDA::lacZ vector	This study
SL1344(*STM0385*) (pDM4-*nadA-pnuC* CDA::lacZ)	SL1344 strain containing pKT100-*STM0385* vector and pDM4-*nadA-pnuC* CDA::lacZ vector	This study
SL1344(*STM3611*) (pDM4-*nadA-pnuC* CDA::lacZ)	SL1344 strain containing pKT100-*STM3611* vector and pDM4-*nadA-pnuC* CDA::lacZ vector	This study
Δ*nadR*(pDM4-*nadA-pnuC* CDA::lacZ)	Δ*nadR* deletion mutant containing pKT100 vector and pDM4-*nadA-pnuC* CDA::lacZ vector	This study
Δ*nadR*(*nadR*) (pDM4-*nadA-pnuC* CDA::lacZ)	Δ*nadR* deletion mutant containing pKT100-*nadR* vector and pDM4-*nadA-pnuC*	This study

	CDA::lacZ vector	
Δ*nadR(STM0385*) (pDM4-*nadA-pnuC* CDA::lacZ)	Δ*nadR* deletion mutant containing pKT100-*STM0385* vector and pDM4-*nadA-pnuC* CDA::lacZ vector	This study
Δ*nadR*(*STM3611*) (pDM4-*nadA-pnuC* CDA::lacZ)	Δ*nadR* deletion mutant containing pKT100-*STM3611* vector and pDM4-*nadA-pnuC* CDA::lacZ vector	This study
nadR(E179A)(pDM4-*nadA-pnuC* CDA::lacZ)	*nadR*(E179A) deletion mutant containing pKT100 vector and pDM4-*nadA-pnuC* CDA::lacZ vector	This study
nadR(E179A)(*STM0385*) (pDM4-*nadA-pnuC* CDA::lacZ)	*nadR*(E179A) deletion mutant containing pKT100-*STM0385* vector and pDM4-*nadA-pnuC* CDA::lacZ vector	This study
nadR(E179A)(*STM3611*) (pDM4-*nadA-pnuC* CDA::lacZ)	*nadR*(E179A) deletion mutant containing pKT100-*STM3611* vector and pDM4-*nadA-pnuC* CDA::lacZ vector	This study
SL1344(pDM4-*nadB* CDA::lacZ)	SL1344 Wild-type strain containing pKT100 vector and pDM4-*nadB* CDA::lacZ vector	This study
SL1344(*STM0385*) (pDM4-*nadB* CDA::lacZ)	SL1344 strain containing pKT100-*STM0385* vector and pDM4-*nadB* CDA::lacZ vector	This study
SL1344(*STM3611*) (pDM4-*nadB* CDA::lacZ)	SL1344 strain containing pKT100-*STM3611* vector and pDM4-*nadB* CDA::lacZ vector	This study
Δ*nadR*(pDM4-*nadB* CDA::lacZ)	Δ*nadR* deletion mutant containing pKT100 vector and pDM4-*nadB* CDA::lacZ vector	This study
Δ*nadR*(*nadR*) (pDM4-*nadB* CDA::lacZ)	Δ*nadR*(*nadR*) deletion mutant containing pKT100-*nadR* vector and pDM4-*nadB* CDA::lacZ vector	This study
Δ*nadR*(*STM0385*) (pDM4-*nadB* CDA::lacZ)	Δ*nadR* deletion mutant containing pKT100-*STM0385* vector and pDM4-*nadB* CDA::lacZ vector	This study
Δ*nadR*(*STM3611*) (pDM4-*nadB* CDA::lacZ)	Δ*nadR* deletion mutant containing pKT100-*STM3611* vector and pDM4-*nadB* CDA::lacZ vector	This study
nadR(E179A)(pDM4-*nadB* CDA::lacZ)	*nadR*(E179A) deletion mutant containing pKT100 vector and pDM4-*nadB* CDA::lacZ vector	This study
nadR(E179A) (*STM0385*) (pDM4-*nadB* CDA::lacZ)	*nadR*(E179A) deletion mutant containing pKT100-*STM0385* vector and pDM4-*nadB* CDA::lacZ vector	This study
nadR(E179A) (*STM3611*) (pDM4-*nadB* CDA::lacZ)	*nadR*(E179A) deletion mutant containing pKT100-*STM3611* vector and pDM4-*nadB* CDA::lacZ vector	This study
HA-*nadR*	SL1344 Wild-type strain expressing in situ tagged HA-*nadR*	This study

HA-*nadR*$_{E179A}$	SL1344 Wild-type strain expressing in situ tagged HA-*nadR*$_{E179A}$	This study
**Plasmids**		
pCas	Crisper-Cas9 system plasmid used for in-frame deletion Km^r	This study
pTargetF1	pTargetF1 with the spectinomycin resistance gene replaced by a chloramphenicol resistance gene Cm^r	This study
pTargetF1-Δ*nadR*	pTargetF1 derivative for *nadR* deletion in SL1344	This study
pTargetF1-HA-*nadR*	pTargetF1 derivative for in situ tagged HA-*nadR* in SL1344	This study
pTargetF1-HA-*nadR*(E179A)	pTargetF1 derivative for in situ tagged HA-*nadR*(E179A) in SL1344	This study
pKT100	Cloning vector p15A replicon Km^r	Laboratory stock
pKT100-*nadR*	*nadR* cloned into pKT100 for complementation	This study
pKT100-*STM0385*	*STM0385* cloned into pKT100 for complementation	This study
pKT100-*STM3611*	*STM3611* cloned into pKT100 for complementation	This study
pDM4	Promoter fusion vector pir sacB Cm^r	Laboratory stock
pDM4-*nadA-pnuC* CDA::lacZ	pDM4 derivative for *nadA-pnuC* promoter fusion	This study
pDM4-*nadB* CDA::lacZ	pDM4 derivative for *nadB* promoter fusion	This study
pET28a	Expression vector with N-terminal hexahistidine affinity tag Km^r	Laboratorys tock
pET28a-*nadR*	pET-28a expressing *nadR*	This study
pET28a-*nadR*$_{K72A}$	pET-28a expressing *nadR*$_{K72A}$	This study
pET28a-*nadR*$_{H80A}$	pET-28a expressing *nadR*$_{H80A}$	This study
pET28a-*nadR*$_{E179A}$	pET-28a expressing *nadR*$_{E179A}$	This study
pET28a-*nadR*$_{R212A}$	pET-28a expressing *nadR*$_{R212A}$	This study
pET28a-*nadR*$_{H80A/R212A}$	pET-28a expressing *nadR*$_{H80A/R212A}$	This study
pET28a-*ycg*R	pET-28a expressing *ycgR*	This study
pET28a-*invF*	pET-28a expressing *invF*	This study
pET28a-*b4390*	pET-28a expressing *b4390*	This study
pET28a-*YPK3620*	pET-28a expressing *YPK3620*	This study
pET28a-*B1H58_03905*	pET-28a expressing *B1H58_03905*	This study
pET28a-*STM0606*	pET-28a expressing *STM0606*	This study
pET28a-*STM2839*	pET-28a expressing *STM2839*	This study
pET28a-*STM2759*	pET-28a expressing *STM2759*	This study

pET28a-*STM2180*	pET-28a expressing *STM2180*	This study
pET28a-*STM2345*	pET-28a expressing *STM2345*	This study
pET28a-*STM4417*	pET-28a expressing *STM4417*	This study
pET28a-*STM2982*	pET-28a expressing *STM2982*	This study
pET28a-*STM4025*	pET-28a expressing *STM4025*	This study
pET28a-*STM3667*	pET-28a expressing *STM3667*	This study
pET28a-*STM0344*	pET-28a expressing *STM0344*	This study
pET28a-*STM1122*	pET-28a expressing *STM1122*	This study
pET28a-*STM3452*	pET-28a expressing *STM3452*	This study
pET28a-*STM4297*	pET-28a expressing *STM4297*	This study
pET28a-*STM2794*	pET-28a expressing *STM2794*	This study
pET28a-*STM3607*	pET-28a expressing *STM3607*	This study
pET28a-*STM1523*	pET-28a expressing *STM1523*	This study
pET28a-*STM3340*	pET-28a expressing *STM3340*	This study
pET28a-*STM4511*	pET-28a expressing *STM4511*	This study
pET28a-*STM1625*	pET-28a expressing *STM1625*	This study
pET28a-*STM3121*	pET-28a expressing *STM3121*	This study
pET28a-*STM2875*	pET-28a expressing *STM2875*	This study
pET28a-*STM2145*	pET-28a expressing *STM2145*	This study
pET28a-*STM2420*	pET-28a expressing *STM2420*	This study
pET28a-*STM3794*	pET-28a expressing *STM3794*	This study
pET28a-*STM0415*	pET-28a expressing *STM0415*	This study
pET28a-*STM0859*	pET-28a expressing *STM0859*	This study
pET28a-*STM2201*	pET-28a expressing *STM2201*	This study
pET28a-*STM2195*	pET-28a expressing *STM2195*	This study
pET28a-*STM3678*	pET-28a expressing *STM3678*	This study
pET28a-*STM2292*	pET-28a expressing *STM2292*	This study
pET28a-*STM1899*	pET-28a expressing *STM1899*	This study
pET28a-*STM0210*	pET-28a expressing *STM0210*	This study
pET28a-*STM0552*	pET-28a expressing *STM0552*	This study
pET28a-*STM2275*	pET-28a expressing *STM2275*	This study
pET28a-*STM3012*	pET-28a expressing *STM3012*	This study
pET28a-*STM0446*	pET-28a expressing *STM0446*	This study
pET28a-*STM3830*	pET-28a expressing *STM3830*	This study
pET28a-*STM2281*	pET-28a expressing *STM2281*	This study
pET28a-*STM3687*	pET-28a expressing *STM3687*	This study
pET28a-*STM0410*	pET-28a expressing *STM0410*	This study
pET28a-*STM1355*	pET-28a expressing *STM1355*	This study
pET28a-*STM3163*	pET-28a expressing *STM3163*	This study
pET28a-*STM3011*	pET-28a expressing *STM3011*	This study
pET28a-*STM1555*	pET-28a expressing *STM1555*	This study
pET28a-*STM3860*	pET-28a expressing *STM3860*	This study
pET28a-*STM1510*	pET-28a expressing *STM1510*	This study
pET28a-*STM2912*	pET-28a expressing *STM2912*	This study

pET28a-*STM3466*	pET-28a expressing *STM3466*	This study
pET28a-*STM2867*	pET-28a expressing *STM2867*	This study
pET28a-*STM1429*	pET-28a expressing *STM1429*	This study
pET28a-*STM4117*	pET-28a expressing *STM4117*	This study
pET28a-*STM0764*	pET-28a expressing *STM0764*	This study
pET28a-*STM4423*	pET-28a expressing *STM4423*	This study
pET28a-*STM0563*	pET-28a expressing *STM0563*	This study
pET28a-*STM4270*	pET-28a expressing *STM4270*	This study
pET28a-*STM2436*	pET-28a expressing *STM2436*	This study
pET28a-*STM3964*	pET-28a expressing *STM3964*	This study
pET28a-*STM3602*	pET-28a expressing *STM3602*	This study
pET28a-*STM1279*	pET-28a expressing *STM1279*	This study
pET28a-*STM0952*	pET-28a expressing *STM0952*	This study
pET28a-*STM1924*	pET-28a expressing *STM1924*	This study
pET28a-*STM2330*	pET-28a expressing *STM2330*	This study
pET28a-*STM2191*	pET-28a expressing *STM2191*	This study
pET28a-*STM3790*	pET-28a expressing *STM3790*	This study
pET28a-*STM3693*	pET-28a expressing *STM3693*	This study
pET28a-*STM0256*	pET-28a expressing *STM0256*	This study
pET28a-*STM0869*	pET-28a expressing *STM0869*	This study
pET28a-*STM3547*	pET-28a expressing *STM3547*	This study
pET28a-*STM0164*	pET-28a expressing *STM0164*	This study
pET28a-*STM3358*	pET-28a expressing *STM3358*	This study
pET28a-*STM3533*	pET-28a expressing *STM3533*	This study
pET28a-*STM0020*	pET-28a expressing *STM0020*	This study
pET28a-*STM3014*	pET-28a expressing *STM3014*	This study
pET28a-*STM3848*	pET-28a expressing *STM3848*	This study
pET28a-*STM2749*	pET-28a expressing *STM2749*	This study
pET28a-*STM2454*	pET-28a expressing *STM2454*	This study
pET28a-*STM1444*	pET-28a expressing *STM1444*	This study
pET28a-*STM1713*	pET-28a expressing *STM1713*	This study
pET28a-*STM0549*	pET-28a expressing *STM0549*	This study
pET28a-*STM2414*	pET-28a expressing *STM2414*	This study
pET28a-*STM3064*	pET-28a expressing *STM3064*	This study
pET28a-*STM0430*	pET-28a expressing *STM0430*	This study
pET28a-*STM4094*	pET-28a expressing *STM4094*	This study
pET28a-*STM4507*	pET-28a expressing *STM4507*	This study
pET28a-*STM0104*	pET-28a expressing *STM0104*	This study
pET28a-*STM4099*	pET-28a expressing *STM4099*	This study
pET28a-*STM3785*	pET-28a expressing *STM3785*	This study
pET28a-*STM3584*	pET-28a expressing *STM3584*	This study
pET28a-*STM4402*	pET-28a expressing *STM4402*	This study
pET28a-*STM4295*	pET-28a expressing *STM4295*	This study
pET28a-*STM3522*	pET-28a expressing *STM3522*	This study

pET28a-*STM1683*	pET-28a expressing *STM1683*	This study
pET28a-*STM2785*	pET-28a expressing *STM2785*	This study
pET28a-*STM0456*	pET-28a expressing *STM0456*	This study
pET28a-*STM2160*	pET-28a expressing *STM2160*	This study
pET28a-*STM2544*	pET-28a expressing *STM2544*	This study
pET28a-*STM2866*	pET-28a expressing *STM2866*	This study
pET28a-*STM0029*	pET-28a expressing *STM0029*	This study
pET28a-*STM1842*	pET-28a expressing *STM1842*	This study
pET28a-*STM3778*	pET-28a expressing *STM3778*	This study
pET28a-*STM3124*	pET-28a expressing *STM3124*	This study
pET28a-*STM3759*	pET-28a expressing *STM3759*	This study
pET28a-*STM0634*	pET-28a expressing *STM0634*	This study
pET28a-*STM4073*	pET-28a expressing *STM4073*	This study
pET28a-*STM1547*	pET-28a expressing *STM1547*	This study
pET28a-*STM1541*	pET-28a expressing *STM1541*	This study
pET28a-*STM2644*	pET-28a expressing *STM2644*	This study
pET28a-*STM1575*	pET-28a expressing *STM1575*	This study
pET28a-*STM0693*	pET-28a expressing *STM0693*	This study
pET28a-*STM4381*	pET-28a expressing *STM4381*	This study
pET28a-*STM1805*	pET-28a expressing *STM1805*	This study
pET28a-*STM1677*	pET-28a expressing *STM1677*	This study
pET28a-*STM3876*	pET-28a expressing *STM3876*	This study
pET28a-*STM2919*	pET-28a expressing *STM2919*	This study
pET28a-*STM4068*	pET-28a expressing *STM4068*	This study
pET28a-*STM3897*	pET-28a expressing *STM3897*	This study
pET28a-*STM3800*	pET-28a expressing *STM3800*	This study
pET28a-*STM3084*	pET-28a expressing *STM3084*	This study
pET28a-*STM1618*	pET-28a expressing *STM1618*	This study
pET28a-*STM1265*	pET-28a expressing *STM1265*	This study
pET28a-*STM3696*	pET-28a expressing *STM3696*	This study
pET28a-*STM4042A*	pET-28a expressing *STM4042A*	This study
pET28a-*STM4266*	pET-28a expressing *STM4266*	This study
pET28a-*STM4241*	pET-28a expressing *STM4241*	This study
pET28a-*STM4548*	pET-28a expressing *STM4548*	This study
pET28a-*STM1691*	pET-28a expressing *STM1691*	This study
pET28a-*STM3025*	pET-28a expressing *STM3025*	This study
pET28a-*STM1488*	pET-28a expressing *STM1488*	This study
pET28a-*STM0835*	pET-28a expressing *STM0835*	This study
pET28a-*STM0692*	pET-28a expressing *STM0692*	This study
pET28a- *STM0014*	pET-28a expressing *STM 0014*	This study
pET28a-*STM1660*	pET-28a expressing *STM1660*	This study
pET28a-*STM4315*	pET-28a expressing *STM4315*	This study
pET28a-*STM0109*	pET-28a expressing *STM 0109*	This study
pET28a-*STM0030*	pET-28a expressing *STM0030*	This study

pET28a-*STM3908*	pET-28a expressing *STM3908*	This study
pET28a-*STM0864*	pET-28a expressing *STM0864*	This study
pET28a-*STM3543*	pET-28a expressing *STM3543*	This study
pET28a-*STM4586*	pET-28a expressing *STM4586*	This study
pET28a-*STM1437*	pET-28a expressing *STM1437*	This study
pET28a-*STM2575*	pET-28a expressing *STM2575*	This study
pET28a-*STM4125*	pET-28a expressing *STM4125*	This study
pET28a-*STM1315*	pET-28a expressing *STM1315*	This study
pET28a-*STM2572*	pET-28a expressing *STM2572*	This study
pET28a-*STM3215*	pET-28a expressing *STM3215*	This study
pET28a-*STM0354*	pET-28a expressing *STM0354*	This study
pET28a-*STM3175*	pET-28a expressing *STM3175*	This study
pET28a-*STM2265*	pET-28a expressing *STM2265*	This study
pET28a-*STM3262*	pET-28a expressing *STM3262*	This study
pET28a-*STM3245*	pET-28a expressing *STM3245*	This study
pET28a-*STM1704*	pET-28a expressing *STM1704*	This study
pET28a-*STM2424*	pET-28a expressing *STM2424*	This study
pET28a-*STM1588*	pET-28a expressing *STM1588*	This study
pET28a-*STM0115*	pET-28a expressing *STM0115*	This study
pET28a-*STM4455*	pET-28a expressing *STM4455*	This study
pET28a-*STM4322*	pET-28a expressing *STM4322*	This study
pET28a-*STM3357*	pET-28a expressing *STM3357*	This study
pET28a-*STM4481*	pET-28a expressing *STM4481*	This study
pET28a-*STM4049*	pET-28a expressing *STM4049*	This study
pET28a-*STM3736*	pET-28a expressing *STM3736*	This study
pET28a-*STM1127*	pET-28a expressing *STM1127*	This study
pET28a-*STM0333*	pET-28a expressing *STM0333*	This study

Table S3. Primers used in this study.

Primiers	5'-3'sequence*	Function
nadR-F	GGACAGCAAATGGGTCGCGGATCCGAATTCGTGTCATCGTTCGACTATCTCAAA	To generate pET28a-*nadR*
nadR-R	GTGGTGGTGCTCGAGTGCGGCCGCAAGCTTTTATCCCTGCTCGCCCAT	
ycgR-F	GGACAGCAAATGGGTCGCGGATCCGAATTCGTGAGTGGTTACAATGAGCAGTTCC	To generate pET28a-*ycgR*
ycgR-R	GTGGTGGTGCTCGAGTGCGGCCGCAAGCTTTTATTCTCGCACTTTATTCGCTCTT	
InvF-F	GGACAGCAAATGGGTCGCGGATCCGAATTCATGTCATTTTCTGAAAGCCGAC	To generate pET28a-*InvF*
InvF-R	GTGGTGGTGCTCGAGTGCGGCCGCAAGCTTTCATTTGTCTGCCAATTGAATAA	
$P_{nadA\text{-}pnuC}$::lacZ-F	ACGCGTCGACCCTGAATGATTGAAGCAGTAACC	To generate pDM4-*nadA-pnuC*
$P_{nadA\text{-}pnuC}$::lacZ-R	CTAGTCTAGATTGTGGGTCAAACATTACGCT	
P_{nadB}::lacZ-F	ACGCGTCGACCCGAGGTAATGTCTCCCC	To generate pDM4-*nadB*
P_{nadB}::lacZ-R	CTAGTCTAGAAGTTGTCATCATGGTTCTTGTTT	
$P_{nadA\text{-}pnuC}$-EMSA-F	ATCAGGCCTACAGACGGCA	EMSA
$P_{nadA\text{-}pnuC}$-EMSA-R	GCTCATCGTGGCTTCTCGT	
$P_{nadA\text{-}pnuC}$-EMSA-CK-F	CGACGACGAAAAGCAATTTTAT	To generate fragment of negative control for EMSA
$P_{nadA\text{-}pnuC}$-EMSA-CK-R	AACCGTACGCCCGCTACC	
P_{nadB}-EMSA-F	GAGTAACCTAACAGCATCTTTATTTC	EMSA
P_{nadB}-EMSA-R	GGTTCTTGTTTAATTTACTAAACATG	
P_{nadB}-EMSA-CK-F	ACTAATCTGTCGCTAAAGCACTC	To generate fragment of negative
P_{nadB}-EMSA-CK-	GCGGATTGTATTACCGAAGA	

R		control for EMSA
Δ*nadR*-sg20(1)-F	AGCTAGCTCAGTCCTAGGTATAATACTAGTAGAAGGGAATACAGCCGAGCGTTTTAGAGCTAGAAATAGC	
Δ*nadR*-sg20(2)-F	AGCTAGCTCAGTCCTAGGTATAATACTAGTCGTGCGTACCGTCGCGATTCGTTTTAGAGCTAGAAATAGC	
Δ*nadR*-sg20-R	TTCAAAAAAAGCACCGACTCGG	To generate pTargetF1-Δ*nadR*
Δ*nadR*-up-F	CCGAGTCGGTGCTTTTTTTGAAGTGGTGTTTGGCGAAGTG	
Δ*nadR*-up-R	AGTGCAACCTTGCTGCTTA	
Δ*nadR*-down-F	TAAGCAGCAAGGTTGCACTTTCAGTGGATCGCAAAGC	
Δ*nadR*-down-R	GGTAATAGATCTAAGCTTCTGCAGGTCGACTATCGCCACCCACATTCT	
pKT100-*nadR*-F	CGCGGATCCGTGTCATCGTTCGACTATCTCAAA	To generate pKT100-*nadR*
pKT100-*nadR*-R	CCGGAATTCTTATCCCTGCTCGCCCAT	
pKT100-*STM0385*-F	CGCGGATCCATGTTCCCAAAAATAATGAATGA	To generate pKT100-*STM0385*
pKT100-*STM0385*-R	ACGCGTCGACTCATGCCGCCACTTCGG	
pKT100-*STM3611*-F	CGCGGATCCATGATAAAGCAGGTTATCCAGCAGCT	To generate pKT100-*STM3611*
pKT100-*STM3611*-R	CCGGAATTCTTACAGGGTCAGAATCACCTCTTCG	
HA-*nadR*-sg20-F	AGCTAGCTCAGTCCTAGGTATAATACTAGTCAGCAAGGTTGCACTTTCAGGTTTTAGAGCTAGAAATAGC	
HA-*nadR*-sg20-R	TTCAAAAAAAGCACCGACTCGG	To generate pTargetF1-HA-*nadR*
HA-*nadR*-up-F	CCGAGTCGGTGCTTTTTTTGAA AGGAATGCTGGTGTTCGGTGT	
HA-*nadR*-up-R	AAGAGCCTCCTGCTGGAAAAATTTGAGATAGTCGAACGATGACAC	
HA-*nadR*-down-F	GTGTACCCATACGATGTTCCAGATTACGCTTCATCGTTCGACTATCTCAA	
HA-*nadR*-down-R	GGTAATAGATCTAAGCTTCTGCAGGTCGACTTATCCCTGCTCGCCCAT	

HA-*nadR*(E179A)-down-R	GGTAATAGATCTAAGCTTCTGCAGGTCGACTTAAGCGTAATCTGGAACATCGTATGGGTATCCCTGCTCGCCCATC	To generate pTargetF1-HA-*nadR*(E179A)
nadR(K72A)-UP-F	GGACAGCAAATGGGTCGCGGATCCGAATTCGTGTCATCGTTCGACTATCTCAA	To generate pET28a-nadR(K72A)
nadR(K72A)-UP-R	CAATGGATAAAATGCACCGAACA	
nadR(K72A)-Down-F	TGTTCGGTGCATTTTATCCATTG	
nadR(K72A)-Down-R	GTGGTGGTGCTCGAGTGCGGCCGCAAGCTTTTATCCCTGCTCGCCCAT	
nadR(H80A)-UP-F	GGACAGCAAATGGGTCGCGGATCCGAATTCGTGTCATCGTTCGACTATCTCAAAAC	To generate pET28a-nadR(H80A)
nadR(H80A)-UP-R	CAAGTAGATTGCTCCGGTATGC	
nadR(H80A)-Down-F	GCATACCGGAGCAATCTACTTG	
nadR(H80A)-Down-R	GTGGTGGTGCTCGAGTGCGGCCGCAAGCTTTTATCCCTGCTCGCCCATC	
nadR(R212A)-UP-F	GGACAGCAAATGGGTCGCGGATCCGAATTCGTGTCATCGTTCGACTATCTCAAAACCG	To generate pET28a-nadR(R212A)
nadR(R212A)-UP-R	ACGGATTTTCTGCGATTTGCGC	
nadR(R212A)-Down-F	GCGCAAATCGCAGAAAATCCGT	
nadR(R212A)-Down-R	GTGGTGGTGCTCGAGTGCGGCCGCAAGCTTTTATCCCTGCTCGCCCATCATCT	
nadR(E179A)-UP-F	GGACAGCAAATGGGTCGCGGATCCGAATTCGTGTCATCGTTCGACTATCTCAAAAC	To generate pET28a-nadR(E179A)
nadR(E179A)-UP-R	TCAGCCTCTGCGGAAGTGTAGATC	
nadR(E179A)-Down-F	GATCTACACTTCCGCAGAGGCTGA	
nadR(E179A)-Down-R	GTGGTGGTGCTCGAGTGCGGCCGCAAGCTTTTATCCCTGCTCGCCCATC	
16S *rRNA*-RT-F	GAGCAAGCGGACCTCATAAAG	qRT-PCR
16S *rRNA*-RT-R	CGTATTCACCGTGGCATTCTG	
nadA-RT-F	CACCTCGGCTGCTGTTAAAG	qRT-PCR
nadA-RT-R	CACGATACACGCTCCTTGC	

nadB-RT-F	CTGGAATCGCAATAAAGAGTGG	qRT-PCR
nadB-RT-R	ATTAGCGACCCGACAGCCT	
pnuC-RT-F	GATGGTGCTGTCTATCGTGG	qRT-PCR
pnuC-RT-R	GTCAGGATCAGATATTCCAGCG	
nadA-*pnuC*-CHIP-F	GTAGGCCAGATAAGACGTTACG	CHIP-qPCR
nadA-*pnuC*-CHIP-R	GCTCATCGTGGCTTCTCGT	
nadB-CHIP-F	GCTAACACCCTGCCCTATAAA	CHIP-qPCR
nadB-CHIP-R	TTCAGGAGTTGTCATCATGGTTCT	
b4390-F	GGACAGCAAATGGGTCGCGGATCCGAATTCATGTCGTCATTTGATTACCTGAA	To generate pET28a-*b4390*
b4390-R	GTGGTGGTGCTCGAGTGCGGCCGCAAGCTTTTATCTCTGCTCCCCCATCAT	
YPK3620-F	GGACAGCAAATGGGTCGCGGATCCGAATTCATGCTGCAGTTCGACTATCTCA	To generate pET28a-*YPK3620*
YPK3620-R	GTGGTGGTGCTCGAGTGCGGCCGCAAGCTTTTATCGCGCATCCGTTAATAC	
B1H58-F	GGACAGCAAATGGGTCGCGGATCCGAATTCATGTCATCATTTGAATATTTAAAAACCG	To generate pET28a-*B1H58*
B1H58-R	GTGGTGGTGCTCGAGTGCGGCCGCAAGCTTTGAAATATAGCCATCAGCCGC	
STM0606-F	GGACAGCAAATGGGTCGCGGATCCGAATTCATGGCTAACCTTTACGACCTT	To generate pET28a-*STM0606*
STM0606-R	GTGGTGGTGCTCGAGTGCGGCCGCAAGCTTTTAATGTTTTTTTGTGATGTGCT	
STM2839-F	GGACAGCAAATGGGTCGCGGATCCGAATTCATGAGTTTTTCCGTGGAGGT	To generate pET28a-*STM2839*
STM2839-R	GTGGTGGTGCTCGAGTGCGGCCGCAAGCTTCTAATCCTTCAGTCCCAGACG	
STM2759-F	GGACAGCAAATGGGTCGCGGATCCGAATTCATGAGTATCTTCGAGGCGC	To generate pET28a-*STM2759*
STM2759-R	GTGGTGGTGCTCGAGTGCGGCCGCAAGCTTTTATTCGAGCCAAAGTTGG	

STM2180-F	GGACAGCAAATGGGTCGCGGATCCGAATTCATGGCGAACTGGGCGC	To generate pET28a-*STM2180*
STM2180-R	GTGGTGGTGCTCGAGTGCGGCCGCAAGCTTCTAAACCTGGGCTAAAAAATACAAA	
STM2345-F	GGACAGCAAATGGGTCGCGGATCCGAATTCATGTCGATACCCCGTAAACG	To generate pET28a-*STM2345*
STM2345-R	GTGGTGGTGCTCGAGTGCGGCCGCAAGCTTTTAGCAGCTTTCCCGCATA	
STM4417-F	GGACAGCAAATGGGTCGCGGATCCGAATTCATGTCTAAACATCAAACTCAACTTTCCTT	To generate pET28a-*STM4417*
STM4417-R	GTGGTGGTGCTCGAGTGCGGCCGCAAGCTTTTACTCCGTCGCCAGCGC	
STM2982-F	GGACAGCAAATGGGTCGCGGATCCGAATTCATGTCAAAACGATTACCTCCTT	To generate pET28a-*STM2982*
STM2982-R	GTGGTGGTGCTCGAGTGCGGCCGCAAGCTTTTATTGTTCGTAACGAAAACG	
STM4025-F	GGACAGCAAATGGGTCGCGGATCCGAATTCATGAGTCTTACCGAACTGACCG	To generate pET28a-*STM4025*
STM4025-R	GTGGTGGTGCTCGAGTGCGGCCGCAAGCTTTCACGCTGAAACGGCGT	
STM3667-F	GGACAGCAAATGGGTCGCGGATCCGAATTCATGAGCCAGAACAACGATAAAGAGA	To generate pET28a-*STM3667*
STM3667-R	GTGGTGGTGCTCGAGTGCGGCCGCAAGCTTTTATTCGCGAACGGTAAATCC	
STM0344-F	GGACAGCAAATGGGTCGCGGATCCGAATTCATGAGTCAGAACAATTATCTGATTGA	To generate pET28a-*STM0344*
STM0344-R	GTGGTGGTGCTCGAGTGCGGCCGCAAGCTTTTAATAACGTTCTTTTATGGAAATG	
STM1122-F	GGACAGCAAATGGGTCGCGGATCCGAATTCATGTCGCAACGCACAGAG	To generate pET28a-*STM1122*
STM1122-R	GTGGTGGTGCTCGAGTGCGGCCGCAAGCTTTTAACGCACGCGAATCC	
STM3452-F	GGACAGCAAATGGGTCGCGGATCCGAATTCATGTCCAGGTCGCTTTTAAC	To generate pET28a-*STM3452*
STM3452-R	GTGGTGGTGCTCGAGTGCGGCCGCAAGCTTTTATTTATCCTGCCCCTGAA	
STM4297-F	GGACAGCAAATGGGTCGCGGATCCGAATTCATGAGTACGCAAGCAATATCCCT	To generate pET28a-*STM4297*
STM4297-R	GTGGTGGTGCTCGAGTGCGGCCGCAAGCTTTTAGCCGGGCATCGTCTG	

STM2794-F	GGACAGCAAATGGGTCGCGGATCC<u>GAATTC</u>ATGACCGCCCTTTCCCA	To generate pET28a-*STM2794*
STM2794-R	GTGGTGGTGCTCGAGTGCGGCCGC<u>AAGCTT</u>CTACATTTTCCCTGCCATCG	
STM3607-F	GGACAGCAAATGGGTCGCGGATCC<u>GAATTC</u>ATGGATAAAATATATGCAATGAAAT	To generate pET28a-*STM3607*
STM3607-R	GTGGTGGTGCTCGAGTGCGGCCGC<u>AAGCTT</u>CTACTCTGCGGCCTCTTTAA	
STM1523-F	GGACAGCAAATGGGTCGCGGATCCGAATTCATGGACCTGACGCAACTGGA	To generate pET28a-*STM1523*
STM1523-R	GTGGTGGTGCTCGAGTGCGGCCGC<u>AAGCTT</u>TCAGTCGGTGCTCGCCAG	
STM3340-F	GGACAGCAAATGGGTCGCGGATCC<u>GAATTC</u>ATGGACGTTATGAACGCATT	To generate pET28a-*STM3340*
STM3340-R	GTGGTGGTGCTCGAGTGCGGCCGC<u>AAGCTT</u>CTACCGCATTTTTTGACTTTT	
STM4511-F	GGACAGCAAATGGGTCGCGGATCC<u>GAATTC</u>ATGGATGTAACTGGAGCAGGTTT	To generate pET28a-*STM4511*
STM4511-R	GTGGTGGTGCTCGAGTGCGGCCGC<u>AAGCTT</u>TTACAGCGCGGCCTGAA	
STM1625-F	GGACAGCAAATGGGTCGCGGATCC<u>GAATTC</u>ATGGAAAAAAATGGTCTGTTTAGTC	To generate pET28a-*STM1625*
STM1625-R	GTGGTGGTGCTCGAGTGCGGCCGC<u>AAGCTT</u>TCACAGCGGCATCGATTTAC	
STM3121-F	GGACAGCAAATGGGTCGCGGATCCGAATTCATGGAACTGCGCCATATTC	To generate pET28a-*STM3121*
STM3121-R	GTGGTGGTGCTCGAGTGCGGCCGC<u>AAGCTT</u>TCAGCCAGTGACTTTCAGGA	
STM2875-F	*GGACAGCAAATGGGTCGCGGATCCGAATTCATGGAAAATGTAACCTTTGTAAGTA*	*To generate pET28a-STM2875*
STM2875-R	*GTGGTGGTGCTCGAGTGCGGCCGCAAGCTTTTAATGGTTCGCCATTTTTAT*	
STM2145-F	GGACAGCAAATGGGTCGCGGATCC<u>GAATTC</u>ATGGAACAAGCGCATACC	To generate pET28a-*STM2145*
STM2145-R	GTGGTGGTGCTCGAGTGCGGCCGC<u>AAGCTT</u>CTACTCCTCGCACACAAAAA	
STM2420-F	GGACAGCAAATGGGTCGCGGATCC<u>GAATTC</u>ATGGAACGAGCCCATCGT	To generate pET28a-*STM2420*
STM2420-R	GTGGTGGTGCTCGAGTGCGGCCGC<u>AAGCTT</u>TTATACCGTCAACGCCGC	
STM3794-F	GGACAGCAAATGGGTCGCGGATCC<u>GAATTC</u>ATGGAGACCAAGCAAAAAGAAC	To generate

STM3794-R	GTGGTGGTGCTCGAGTGCGGCCGCAAGCTTTTATTCA CTTTCCGAATCAGGC	pET28a-*STM3794*
STM0415-F	GGACAGCAAATGGGTCGCGGATCCGAATTCATGCATT GCCCATTTTGTT	To generate
STM0415-R	GTGGTGGTGCTCGAGTGCGGCCGCAAGCTTTTAGTC CTGTAGGCGAGCG	pET28a-*STM0415*
STM0859-F	GGACAGCAAATGGGTCGCGGATCCGAATTCATGCACT TTGATATAAAAGATTTAAAAT	To generate
STM0859-R	GTGGTGGTGCTCGAGTGCGGCCGCAAGCTTTCACTC AGTCTGAGCTGTGAGC	pET28a-*STM0859*
STM2201-F	GGACAGCAAATGGGTCGCGGATCCGAATTCATGCATA TTACGCTACGACAACTT	To generate
STM2201-R	GTGGTGGTGCTCGAGTGCGGCCGCAAGCTTTTATTCA CAGTAACTCAAAAAACGC	pET28a-*STM2201*
STM2195-F	GGACAGCAAATGGGTCGCGGATCCGAATTCGTGAAAT ACAATACGATGAATAACG	To generate
STM2195-R	GTGGTGGTGCTCGAGTGCGGCCGCAAGCTTTCACCA ACATAATGAATTTTCC	pET28a-*STM2195*
STM3678-F	GGACAGCAAATGGGTCGCGGATCCGAATTCATGCTCG AATTATCCATAACATTA	To generate
STM3678-R	GTGGTGGTGCTCGAGTGCGGCCGCAAGCTTTCAAGA ATTAACGTGTTCCTTACT	pET28a-*STM3678*
STM2292-F	*GGACAGCAAATGGGTCGCGGATCCGAATTCATGTTGG AATCAAGTAAAGTCCC*	*To generate*
STM2292-R	*GTGGTGGTGCTCGAGTGCGGCCGCAAGCTTTTAAGAT GCGAGTGAGTCGAAG*	*pET28a-STM2292*
STM1899-F	*GGACAGCAAATGGGTCGCGGATCCGAATTCATGGCAG GTCATAGTAAATGGGC*	*To generate*
STM1899-R	GTGGTGGTGCTCGAGTGCGGCCGCAAGCTTTTACAG GGTCGCCGCCAC	*pET28a-STM1899*
STM0210-F	GGACAGCAAATGGGTCGCGGATCCGAATTCATGGCTG GCTGGCATCTT	To generate
STM0210-R	GTGGTGGTGCTCGAGTGCGGCCGCAAGCTTTTAACG CTGTTCATCCAGTTGTA	pET28a-*STM0210*
STM0552-F	GGACAGCAAATGGGTCGCGGATCCGAATTCATGCTGC GTATCGCTATTA	To generate
STM0552-R	GTGGTGGTGCTCGAGTGCGGCCGCAAGCTTTTACTTA CTGAGTAAGAAATGAAGG	pET28a-*STM0552*
STM2275-F	GGACAGCAAATGGGTCGCGGATCCGAATTCATGAAAA ATAGATTAAAGCGTGACTTTAG	To generate
STM2275-R	GTGGTGGTGCTCGAGTGCGGCCGCAAGCTTTTACGC TTTCAGCGCAAGAAG	pET28a-*STM2275*
STM3012-F	GGACAGCAAATGGGTCGCGGATCCGAATTCATGATGG CGACAATGCTGGAT	To generate

STM3012-R	GTGGTGGTGCTCGAGTGCGGCCGCAAGCTTTTAGGC CGGACGTTGAAAAAAC	pET28a-*STM3012*
STM0446-F	GGACAGCAAATGGGTCGCGGATCCGAATTCATGATGA TACGTGAGCAAATAGAAG	To generate
STM0446-R	GTGGTGGTGCTCGAGTGCGGCCGCAAGCTTCTACGC GATGCTTCCCG	pET28a-*STM0446*
STM3830-F	GGACAGCAAATGGGTCGCGGATCCGAATTCATGACTC TCAATAAAACCGATC	To generate
STM3830-R	GTGGTGGTGCTCGAGTGCGGCCGCAAGCTTTCATGT GATTTCCTTTAACCTTC	pET28a-*STM3830*
STM2281-F	GGACAGCAAATGGGTCGCGGATCCGAATTCATGACA CTAACCCAAATCCAC	To generate
STM2281-R	GTGGTGGTGCTCGAGTGCGGCCGCAAGCTTTTATAG CAACCCCTCATCG	pET28a-*STM2281*
STM3687-F	GGACAGCAAATGGGTCGCGGATCCGAATTCATGACG CAGAATCCGGCG	To generate
STM3687-R	GTGGTGGTGCTCGAGTGCGGCCGCAAGCTTTTACTT TTGAAAGGCCTTTTTTAAGC	pET28a-*STM3687*
STM0410-F	GGACAGCAAATGGGTCGCGGATCCGAATTCATGACC AGACGCGCTGACCG	To generate
STM0410-R	GTGGTGGTGCTCGAGTGCGGCCGCAAGCTTTTACG GCATTTTTTCGTGCTCG	pET28a-*STM0410*
STM1355-F	GGACAGCAAATGGGTCGCGGATCCGAATTCATGTATC AGCGCTGTTTTGACT	To generate
STM1355-R	GTGGTGGTGCTCGAGTGCGGCCGCAAGCTTTCAGGT ATTTGGCAGCAAATAC	pET28a-*STM1355*
STM3163-F	GGACAGCAAATGGGTCGCGGATCCGAATTCATGAAAA AATGGCGCGTT	To generate
STM3163-R	GTGGTGGTGCTCGAGTGCGGCCGCAAGCTTTTAGCT CCCTTGCATTGTCC	pET28a-*STM3163*
STM3011-F	GGACAGCAAATGGGTCGCGGATCCGAATTCATGGCG A CCATAAAAGATG	To generate
STM3011-R	GTGGTGGTGCTCGAGTGCGGCCGCAAGCTTATGGC GA CCATAAAAGATG	pET28a-*STM3011*
STM1555-F	GGACAGCAAATGGGTCGCGGATCCGAATTCATGGCG ACAATAAAGGATG	To generate
STM1555-R	GTGGTGGTGCTCGAGTGCGGCCGCAAGCTTTCACC GTGAAAAATAGGG	pET28a-*STM1555*
STM3860-F	GGACAGCAAATGGGTCGCGGATCCGAATTCATGACA ACCCGGCACACTG	To generate
STM3860-R	GTGGTGGTGCTCGAGTGCGGCCGCAAGCTTTTAAG GCCCTGGCTCAATCC	pET28a-*STM3860*

STM1510-F	GGACAGCAAATGGGTCGCGGATCCGAATTCATGACCGTCGAAACGCAGC	To generate
STM1510-R	GTGGTGGTGCTCGAGTGCGGCCGCAAGCTTTTACTCTTCGCTAAACCAGCCG	pET28a-*STM1510*
STM2912-F	GGACAGCAAATGGGTCGCGGATCCGAATTCATGACAGTAAAGGAGCTCGCCATG	To generate
STM2912-R	GTGGTGGTGCTCGAGTGCGGCCGCAAGCTTTACGATGATGGGGCGTGCTC	pET28a-*STM2912*
STM3466-F	GGACAGCAAATGGGTCGCGGATCCGAATTCTGGTGCTTGGCAAACCG	To generate
STM3466-R	GTGGTGGTGCTCGAGTGCGGCCGCAAGCTTAACGGGTGCCGTAGACG	pET28a-*STM3466*
STM2867-F	GGACAGCAAATGGGTCGCGGATCCGAATTCTGGTATTGCCTTCAATGAATA	To generate
STM2867-R	GTGGTGGTGCTCGAGTGCGGCCGCAAGCTTCAATGGTTCATTGTACGCA	pET28a-*STM2867*
STM1429-F	GGACAGCAAATGGGTCGCGGATCCGAATTCTGTGGTCGGAATACTCGCTTGA	To generate
STM1429-R	GTGGTGGTGCTCGAGTGCGGCCGCAAGCTTTAATCCCCGCTGTCGGGA	pET28a-*STM1429*
STM4117-F	GGACAGCAAATGGGTCGCGGATCCGAATTCATGTATCACGACGTCAGCCAC	To generate
STM4117-R	GTGGTGGTGCTCGAGTGCGGCCGCAAGCTTTTATCCTGGAGTAATCTGCTTTTC	pET28a-*STM4117*
STM0764-F	GGACAGCAAATGGGTCGCGGATCCGAATTCGTGTATAATGCTACTTATATAAATGAAAC	To generate
STM0764-R	GTGGTGGTGCTCGAGTGCGGCCGCAAGCTTTATATTTGTTCTTCAACAAAAATTTC	pET28a-*STM0764*
STM4423-F	GGACAGCAAATGGGTCGCGGATCCGAATTCCTTGGCGTATGTGAGTCAGG	To generate
STM4423-R	GTGGTGGTGCTCGAGTGCGGCCGCAAGCTTTTAATATTCAAATAATAAATAATTAGC	pET28a-*STM4423*
STM0563-F	GGACAGCAAATGGGTCGCGGATCCGAATTCCATGGATGCGCTTAGTCGGCTATTG	To generate
STM0563-R	GTGGTGGTGCTCGAGTGCGGCCGCAAGCTTTTATCGTCCGAGCGCGCTGA	pET28a-*STM0563*
STM4270-F	GGACAGCAAATGGGTCGCGGATCCGAATTCCATGGATATCAGAACGCTGC	To generate
STM4270-R	GTGGTGGTGCTCGAGTGCGGCCGCAAGCTTTTACGGTTGTTTCAGCCA	pET28a-*STM4270*
STM2436-F	GGACAGCAAATGGGTCGCGGATCCGAATTCATGATCGACGGAAAAACCGCT	To generate
STM2436-R	GTGGTGGTGCTCGAGTGCGGCCGCAAGCTTTTAGCGATTTAGCGCCTGATG	pET28a-*STM2436*

STM3964-F	GGACAGCAAATGGGTCGCGGATCCGAATTCATGATC GAAATTAAACATCTGAAAACAC	To generate
STM3964-R	GTGGTGGTGCTCGAGTGCGGCCGCAAGCTTTACAG GCGCGGCTGTGAT	pET28a-*STM3964*
STM3602-F	GGACAGCAAATGGGTCGCGGATCCGAATTCATGATCG AGCAACCCGACAGT	To generate
STM3602-R	GTGGTGGTGCTCGAGTGCGGCCGCAAGCTTTCATAC GGTAAACCGTATTTTATCG	pET28a-*STM3602*
STM1279-F	GGACAGCAAATGGGTCGCGGATCCGAATTCATGATTG GTCTGAGACTGGACG	To generate
STM1279-R	GTGGTGGTGCTCGAGTGCGGCCGCAAGCTTTTAATT GTGGGGAAGCAGTAACTC	pET28a-*STM1279*
STM0952-F	GGACAGCAAATGGGTCGCGGATCCGAATTCATGATAA AAACGGATCTCAATG	To generate
STM0952-R	GTGGTGGTGCTCGAGTGCGGCCGCAAGCTTTTACTC TTCCACCATTCGC	pET28a-*STM0952*
STM1924-F	GGACAGCAAATGGGTCGCGGATCCGAATTCATGAGTG AAAAAAGCATTGTTCAG	To generate
STM1924-R	GTGGTGGTGCTCGAGTGCGGCCGCAAGCTTTTAAAC AGCCTGTTCGATCTGT	pET28a-*STM1924*
STM2330-F	GGACAGCAAATGGGTCGCGGATCCGAATTCATGATAA ATGCAAATCGTCCG	To generate
STM2330-R	GTGGTGGTGCTCGAGTGCGGCCGCAAGCTTCTACTC AAAACCCCCTTCCAC	pET28a-*STM2330*
STM2191-F	GGACAGCAAATGGGTCGCGGATCCGAATTCATGATCA CCATTCGTGATGTAG	To generate
STM2191-R	GTGGTGGTGCTCGAGTGCGGCCGCAAGCTTTCAGTT AGTGATCAGTACCGCA	pET28a-*STM2191*
STM3790-F	GGACAGCAAATGGGTCGCGGATCCGAATTCATGATC ACCGTTGCCCT	To generate
STM3790-R	GTGGTGGTGCTCGAGTGCGGCCGCAAGCTTTACCAA CCGTCAAACATG	pET28a-*STM3790*
STM3693-F	GGACAGCAAATGGGTCGCGGATCCGAATTCGTGATT GTGATGCCAAAACGC	To generate
STM3693-R	GTGGTGGTGCTCGAGTGCGGCCGCAAGCTTCATGAT TTATTCTCCCTGGTCATC	pET28a-*STM3693*
STM0256-F	GGACAGCAAATGGGTCGCGGATCCGAATTCATGAAA GCCACCTCCGAAGA	To generate
STM0256-R	GTGGTGGTGCTCGAGTGCGGCCGCAAGCTTTTACCC CTCTTTCACAGCTCCT	pET28a-*STM0256*
STM0869-F	GGACAGCAAATGGGTCGCGGATCCGAATTCATGAAAG AAAATGCCGTAAGACG	To generate
STM0869-R	GTGGTGGTGCTCGAGTGCGGCCGCAAGCTTTCAACC CCGCCATTTCG	pET28a-*STM0869*

STM3547-F	GGACAGCAAATGGGTCGCGGATCCGAATTCATGAAAA ATAATGTATGTGTTATTG	To generate pET28a-*STM3547*
STM3547-R	GTGGTGGTGCTCGAGTGCGGCCGCAAGCTTTTAGTG AGTAGAGATAGTTTGTTTGTA	
STM0164-F	GGACAGCAAATGGGTCGCGGATCCGAATTCATGAAAG GACAACACCGTCTGG	To generate pET28a-*STM0164*
STM0164-R	GTGGTGGTGCTCGAGTGCGGCCGCAAGCTTTCACTC CGGCTGCACCAG TCGATCTGT	
STM3358-F	GGACAGCAAATGGGTCGCGGATCCGAATTCGTGAAAA AGATCCAAAGAACGC	To generate pET28a-*STM3358*
STM3358-R	GTGGTGGTGCTCGAGTGCGGCCGCAAGCTTTTAGGC TTCTGCACTTTCCC	
STM3533-F	GGACAGCAAATGGGTCGCGGATCCGAATTCATGAAAA AGATTACGACATCTATTC	To generate pET28a-*STM3533*
STM3533-R	GTGGTGGTGCTCGAGTGCGGCCGCAAGCTTTCAGCA CATCAGACGCG	
STM0020-F	GGACAGCAAATGGGTCGCGGATCCGAATTCATGAAA AAAATGATGAACGACGCT	To generate pET28a-*STM0020*
STM0020-R	GTGGTGGTGCTCGAGTGCGGCCGCAAGCTTTTAATA CGCCACTGGCAGTTTTC	
STM3014-F	GGACAGCAAATGGGTCGCGGATCCGAATTCATGGCT GCCGTTAATTTACG	To generate pET28a-*STM3014*
STM3014-R	GTGGTGGTGCTCGAGTGCGGCCGCAAGCTTTTATGC TTTCGTCATCGGC	
STM3848-F	GGACAGCAAATGGGTCGCGGATCCGAATTCATGAAGA AATCCCTTACCAATCTCG	To generate pET28a-*STM3848*
STM3848-R	GTGGTGGTGCTCGAGTGCGGCCGCAAGCTTTCAGAT AAGGCGACGGCAGA	
STM2749-F	GGACAGCAAATGGGTCGCGGATCCGAATTCATGAAAA AGTCAACAACCAAAGACTG	To generate pET28a-*STM2749*
STM2749-R	GTGGTGGTGCTCGAGTGCGGCCGCAAGCTTATGTCC CGGAGTTGTCGGT	
STM2454-F	GGACAGCAAATGGGTCGCGGATCCGAATTCATGAAAA AGACCCGTACAGCGAATT	To generate pET28a-*STM2454*
STM2454-R	GTGGTGGTGCTCGAGTGCGGCCGCAAGCTTTCAAGC CCATTGCCGCATC	
STM1444-F	GGACAGCAAATGGGTCGCGGATCCGAATTCATGAAAT TGGAATCGCCACTAGGTT	To generate pET28a-*STM1444*
STM1444-R	GTGGTGGTGCTCGAGTGCGGCCGCAAGCTTTCAATC GTGAGAGTGCAATTCCATAAT	
STM1713-F	GGACAGCAAATGGGTCGCGGATCCGAATTCATGAAAT TGCAGCAGCTTCGT	To generate pET28a-*STM1713*
STM1713-R	GTGGTGGTGCTCGAGTGCGGCCGCAAGCTTTTACTTT TCAGGCAGCTTTATATCC	

STM0549-F	GGACAGCAAATGGGTCGCGGATCCGAATTCATGAAACCTGCATCTGTTATCA	To generate pET28a-*STM0549*
STM0549-R	GTGGTGGTGCTCGAGTGCGGCCGCAAGCTTTTACAATAATTCGTGTGATTTGG	
STM2414-F	GGACAGCAAATGGGTCGCGGATCCGAATTCATGAAAAGATTACGCAGTAAAATGACC	To generate pET28a-*STM2414*
STM2414-R	GTGGTGGTGCTCGAGTGCGGCCGCAAGCTTTTAGTCCGCCTCATTGATACCTAAAC	
STM3064-F	GGACAGCAAATGGGTCGCGGATCCGAATTCATGAAACGTCCGGACTACAGAACAC	To generate pET28a-*STM3064*
STM3064-R	GTGGTGGTGCTCGAGTGCGGCCGCAAGCTTCTAATCCTGACGCAGCACCTTGT	
STM0430-F	GGACAGCAAATGGGTCGCGGATCCGAATTCATGAAATCTATCCCGGGCGAT	To generate pET28a-*STM0430*
STM0430-R	GTGGTGGTGCTCGAGTGCGGCCGCAAGCTTCTAGTGGGTATCTACCTCAATACGCA	
STM4094-F	GGACAGCAAATGGGTCGCGGATCCGAATTCGTGAAATCGAACAAGCAGGTTACTG	To generate pET28a-*STM4094*
STM4094-R	GTGGTGGTGCTCGAGTGCGGCCGCAAGCTTTAGGGTAAAGCGCGCGTAG	
STM4507-F	GGACAGCAAATGGGTCGCGGATCCGAATTCATGAAATCAAACACTTCTCAGCAAAG	To generate pET28a-*STM4507*
STM4507-R	GTGGTGGTGCTCGAGTGCGGCCGCAAGCTTTCAGGCATCAACGTTGTCCAG	
STM0104-F	GGACAGCAAATGGGTCGCGGATCCGAATTCATGGCTGAAACGCAAAATGAT	To generate pET28a-*STM0104*
STM0104-R	GTGGTGGTGCTCGAGTGCGGCCGCAAGCTTTTATTCACATCCGGCCCTG	
STM4099-F	GGACAGCAAATGGGTCGCGGATCCGAATTCATGGCTGAATGGAGCGG	To generate pET28a-*STM4099*
STM4099-R	GTGGTGGTGCTCGAGTGCGGCCGCAAGCTTTCAGTATTCCCACGTTTCCG	
STM3785-F	GGACAGCAAATGGGTCGCGGATCCGAATTCATGAAAACATTGAGTAAAAGTTCACACA	To generate pET28a-*STM3785*
STM3785-R	GTGGTGGTGCTCGAGTGCGGCCGCAAGCTTTCAGGCATCCAGGCGCT	
STM3584-F	GGACAGCAAATGGGTCGCGGATCCGAATTCATGCAACGTGTCACCATCACC	To generate pET28a-*STM3584*
STM3584-R	GTGGTGGTGCTCGAGTGCGGCCGCAAGCTTTTAGTCTTCTTTCGGCAAACACTG	
STM4402-F	GGACAGCAAATGGGTCGCGGATCCGAATTCATGCGCGCTCATACTCTTTCC	To generate pET28a-*STM4402*
STM4402-R	GTGGTGGTGCTCGAGTGCGGCCGCAAGCTTCTAGCCGCCATCTGATAAACG	

STM4295-F	GGACAGCAAATGGGTCGCGGATCCGAATTCATGAGGATTTGCAGCAACGAAC	To generate
STM4295-R	GTGGTGGTGCTCGAGTGCGGCCGCAAGCTTTTAGGCAGCCGATCGTTCC	pET28a-*STM4295*
STM3522-F	GGACAGCAAATGGGTCGCGGATCCGAATTCATGCGAAAAACGGTGGCCTT	To generate
STM3522-R	GTGGTGGTGCTCGAGTGCGGCCGCAAGCTTTTAATTCTGTAAAACGTCCCACGTCA	pET28a-*STM3522*
STM1683-F	GGACAGCAAATGGGTCGCGGATCCGAATTCATGCGTCTGGAAGTCTTTTGTG	To generate
STM1683-R	GTGGTGGTGCTCGAGTGCGGCCGCAAGCTTCTACTCTTCACCCTTCTTCTGGC	pET28a-*STM1683*
STM2785-F	GGACAGCAAATGGGTCGCGGATCCGAATTCATGCGTCTCTTATTGGCAG	To generate
STM2785-R	GTGGTGGTGCTCGAGTGCGGCCGCAAGCTTTTAACCCACTTCATCGCC	pET28a-*STM2785*
STM0456-F	GGACAGCAAATGGGTCGCGGATCCGAATTCATGAGGCTACTGAATCGGCTTAATC	To generate
STM0456-R	GTGGTGGTGCTCGAGTGCGGCCGCAAGCTTTCACGGCGAAGGCGGC	pET28a-*STM0456*
STM2160-F	GGACAGCAAATGGGTCGCGGATCCGAATTCATGGCGCTTTACACAATTGGT	To generate
STM2160-R	GTGGTGGTGCTCGAGTGCGGCCGCAAGCTTTTAAACGCCAAGGGGATGAAT	pET28a-*STM2160*
STM2544-F	GGACAGCAAATGGGTCGCGGATCCGAATTCATGAGACTGACATCTAAAGGGCGT	To generate
STM2544-R	GTGGTGGTGCTCGAGTGCGGCCGCAAGCTTTTAAGCGCGTAATTTAACGTCG	pET28a-*STM2544*
STM2866-F	GGACAGCAAATGGGTCGCGGATCCGAATTCATGAGAAATGTAATTATATACGGTA	To generate
STM2866-R	GTGGTGGTGCTCGAGTGCGGCCGCAAGCTTTTAATTCATTCCTACCGCA	pET28a-*STM2866*
STM0029-F	GGACAGCAAATGGGTCGCGGATCCGAATTCATGAGACAATATACTATTAATAACGAATT	To generate
STM0029-R	GTGGTGGTGCTCGAGTGCGGCCGCAAGCTTTCAGATAAACGGCGCTAAA	pET28a-*STM0029*
STM1842-F	GGACAGCAAATGGGTCGCGGATCCGAATTCATGGCAAACGCAGATCTGG	To generate
STM1842-R	GTGGTGGTGCTCGAGTGCGGCCGCAAGCTTTTAGAAGGGATAATCGTTGTAACC	pET28a-*STM1842*
STM3778-F	GGACAGCAAATGGGTCGCGGATCCGAATTCATGTCAGCAAAAACTAAATTCAAAAGCC	To generate
STM3778-R	GTGGTGGTGCTCGAGTGCGGCCGCAAGCTTTCATACCAGCACCTTTAGCCCGT	pET28a-*STM3778*

STM3124-F	GGACAGCAAATGGGTCGCGGATCCGAATTCATGTCAGCCTTATCAACTACTGGCCATA	To generate
STM3124-R	GTGGTGGTGCTCGAGTGCGGCCGCAAGCTTTCACATATGCTGTGCATGTTGTACCG	pET28a-*STM3124*
STM3759-F	GGACAGCAAATGGGTCGCGGATCCGAATTCATGTTTTGGCATGGTAACAATGC	To generate
STM3759-R	GTGGTGGTGCTCGAGTGCGGCCGCAAGCTTTTACCCATGTGTCACCCCGC	pET28a-*STM3759*
STM0634-F	GGACAGCAAATGGGTCGCGGATCCGAATTCGTGAGTGATAATAATCAGGCAGAAAGAC	To generate
STM0634-R	GTGGTGGTGCTCGAGTGCGGCCGCAAGCTTTCAGAACGCCTCACGGATGA	pET28a-*STM0634*
STM4073-F	GGACAGCAAATGGGTCGCGGATCCGAATTCATGAGCGATAATACGTTGGTATCTG	To generate
STM4073-R	GTGGTGGTGCTCGAGTGCGGCCGCAAGCTTTTATTTTTCAATAATTTGAATTATTTTCC	pET28a-*STM4073*
STM1547-F	GGACAGCAAATGGGTCGCGGATCCGAATTCATGTCAGAAAATAAAAATGTGCAAGAT	To generate
STM1547-R	GTGGTGGTGCTCGAGTGCGGCCGCAAGCTTTTATTCCGGCATTGAAGCATC	pET28a-*STM1547*
STM1541-F	GGACAGCAAATGGGTCGCGGATCCGAATTCATGGTTATACCTGAGGTCAGGCAG	To generate
STM1541-R	GTGGTGGTGCTCGAGTGCGGCCGCAAGCTTTAGCCGCCGACAATCTTTCT	pET28a-*STM1541*
STM2644-F	GGACAGCAAATGGGTCGCGGATCCGAATTCATGGATCTGCGTCGGTTTATTAC	To generate
STM2644-R	GTGGTGGTGCTCGAGTGCGGCCGCAAGCTTTTAACCCGGCATTTTTTTATCTTC	pET28a-*STM2644*
STM1575-F	GGACAGCAAATGGGTCGCGGATCCGAATTCTACCTTAACCGGGATGAACG	To generate
STM1575-R	GTGGTGGTGCTCGAGTGCGGCCGCAAGCTTCAAAGAGTTCAAGCGTAATCATAC	pET28a-*STM1575*
STM0693-F	GGACAGCAAATGGGTCGCGGATCCGAATTCCTGACAACAATACCGCATTAAAG	To generate
STM0693-R	GTGGTGGTGCTCGAGTGCGGCCGCAAGCTTTAGTCGCGTCATCGTGCG	pET28a-*STM0693*
STM4381-F	GGACAGCAAATGGGTCGCGGATCCGAATTCTGAAGCACAAAGACATCAAATACT	To generate
STM4381-R	GTGGTGGTGCTCGAGTGCGGCCGCAAGCTTCGCGGAGTATGCTTACACC	pET28a-*STM4381*
STM1805-F	GGACAGCAAATGGGTCGCGGATCCGAATTCATGGTCATTAAGGCGCAGAGCC	To generate
STM1805-R	GTGGTGGTGCTCGAGTGCGGCCGCAAGCTTTTATCGTCCCTGAATCGCTAAATCG	pET28a-*STM1805*

STM1677-F	GGACAGCAAATGGGTCGCGGATCC<u>GAATTC</u>ATGGACAAGCTGGAAGCGATG	To generate
STM1677-R	GTGGTGGTGCTCGAGTGCGGCCGC<u>AAGCTT</u>TTAGCGAAGAGGCGCCGG	pET28a-*STM1677*
STM3876-F	GGACAGCAAATGGGTCGCGGATCC<u>GAATTC</u>ATGGAAAATTATCAGATCGACA	To generate
STM3876-R	GTGGTGGTGCTCGAGTGCGGCCGC<u>AAGCTT</u>TCATGGCTTGATGGTGC	pET28a-*STM3876*
STM2919-F	GGACAGCAAATGGGTCGCGGATCC<u>GAATTC</u>TTGATACCTACTGAGCGTCGACAA	To generate
STM2919-R	GTGGTGGTGCTCGAGTGCGGCCGC<u>AAGCTT</u>CTATTGTTTGGCCATCAGCAGTG	pET28a-*STM2919*
STM4068-F	GGACAGCAAATGGGTCGCGGATCC<u>GAATTC</u>TTGCAGGTTGATAAAACGAGCTT	To generate
STM4068-R	GTGGTGGTGCTCGAGTGCGGCCGC<u>AAGCTT</u>GATAATGAATTTCACCAAAGTAT	pET28a-*STM4068*
STM3897-F	GGACAGCAAATGGGTCGCGGATCC<u>GAATTC</u>GTGGAGAGCACAGTGGATAC	To generate
STM3897-R	GTGGTGGTGCTCGAGTGCGGCCGC<u>AAGCTT</u>TCATTGTTCATCCAGCACA	pET28a-*STM3897*
STM3800-F	GGACAGCAAATGGGTCGCGGATCC<u>GAATTC</u>ATGGAACCCCTTCGCGATAC	To generate
STM3800-R	GTGGTGGTGCTCGAGTGCGGCCGC<u>AAGCTT</u>TCACACCTGTTCCTGCAACCA	pET28a-*STM3800*
STM3084-F	GGACAGCAAATGGGTCGCGGATCC<u>GAATTC</u>ATGGAACAAATTTTAACCAAGCGG	To generate
STM3084-R	GTGGTGGTGCTCGAGTGCGGCCGC<u>AAGCTT</u>TCAGGTGGCGAAGAAAAAATCATCA	pET28a-*STM3084*
STM1618-F	GGACAGCAAATGGGTCGCGGATCC<u>GAATTC</u>ATGAGCCAACAGCGCCC	To generate
STM1618-R	GTGGTGGTGCTCGAGTGCGGCCGC<u>AAGCTT</u>TTAACGCGCTCCTGATGAGG	pET28a-*STM1618*
STM1265-F	GGACAGCAAATGGGTCGCGGATCC<u>GAATTC</u>GACTGGTTCCGCATCATCT	To generate
STM1265-R	GTGGTGGTGCTCGAGTGCGGCCGC<u>AAGCTT</u>CAATGACCTTACTATTTACACG	pET28a-*STM1265*
STM3696-F	GGACAGCAAATGGGTCGCGGATCC<u>GAATTC</u>GTGAAGAGAACCAAATCACCTCGTG	To generate
STM3696-R	GTGGTGGTGCTCGAGTGCGGCCGC<u>AAGCTT</u>TTATGCGGACTCTCGCCCAATC	pET28a-*STM3696*
STM4042A-F	GGACAGCAAATGGGTCGCGGATCC<u>GAATTC</u>GTGAACGTTGGATGCTTTATGACACA	To generate
STM4042A-R	GTGGTGGTGCTCGAGTGCGGCCGC<u>AAGCTT</u>TCAATTCTGCTCCGCTACCAGGA	pET28a-*STM4042*

		A
STM4266-F	GGACAGCAAATGGGTCGCGGATCCGAATTCATGGAAA<br>AAAAATCTCCCCGTT	To<br>generate
STM4266-R	GTGGTGGTGCTCGAGTGCGGCCGCAAGCTTTTAATC<br>ATCTTCAAGCAGCCGG	pET28a-<br>*STM4266*
STM4241-F	GGACAGCAAATGGGTCGCGGATCCGAATTCATGGAAA<br>AGACCACAACG	To<br>generate
STM4241-R	GTGGTGGTGCTCGAGTGCGGCCGCAAGCTTCTAACG<br>TGGTTTTTTCTTCA	pET28a-<br>*STM4241*
STM4548-F	GGACAGCAAATGGGTCGCGGATCCGAATTCATGGAAA<br>ACAGAATCAGCAAGAG	To<br>generate
STM4548-R	GTGGTGGTGCTCGAGTGCGGCCGCAAGCTTCTATAC<br>CCGATGCTGGAGC	pET28a-<br>*STM4584*
STM1691-F	GGACAGCAAATGGGTCGCGGATCCGAATTCATGGCT<br>GAATTTAAAGATAACC	To<br>generate
STM1691-R	GTGGTGGTGCTCGAGTGCGGCCGCAAGCTTCTAAA<br>GCTGATGCTTTTTAAG	pET28a-<br>*STM1691*
STM3025-F	GGACAGCAAATGGGTCGCGGATCCGAATTCGTGACG<br>ATCGCCGTGGC	To<br>generate
STM3025-R	GTGGTGGTGCTCGAGTGCGGCCGCAAGCTTTTATTG<br>CGGAGCGATAAACG	pET28a-<br>*STM3025*
STM1488-F	GGACAGCAAATGGGTCGCGGATCCGAATTCGTTGCTG<br>ATAGTCAGCCT	To<br>generate
STM1488-R	GTGGTGGTGCTCGAGTGCGGCCGCAAGCTTCCCTGT<br>AATAGACGAATCAA	pET28a-<br>*STM1488*
STM0835-F	GGACAGCAAATGGGTCGCGGATCCGAATTCATGGGTC<br>GTCGCGCAGGT	To<br>generate
STM0835-R	GTGGTGGTGCTCGAGTGCGGCCGCAAGCTTTCATTC<br>TGCAGATGTTCCATGCTG	pET28a-<br>*STM0835*
STM0692-F	GGACAGCAAATGGGTCGCGGATCCGAATTCATGGAG<br>CTTCGCCAGTTAC	To<br>generate
STM0692-R	GTGGTGGTGCTCGAGTGCGGCCGCAAGCTTTTAGTA<br>AGCGTAAAGGGTTGC	pET28a-<br>*STM0692*
STM0014-F	GGACAGCAAATGGGTCGCGGATCCGAATTCATGGGAT<br>CGAAAGGTGCCAAC	To<br>generate
STM0014-R	GTGGTGGTGCTCGAGTGCGGCCGCAAGCTTTTAGCG<br>ACGGAAGGAGGAAAGT	pET28a-<br>*STM0014*
STM1660-F	GGACAGCAAATGGGTCGCGGATCCGAATTCATGTTA<br>AAATTGACAAATATCAATTACGG	To<br>generate
STM1660-R	GTGGTGGTGCTCGAGTGCGGCCGCAAGCTTTTAAGC<br>GACGTTGCGGGT	pET28a-<br>*STM1600*
STM4315-F	GGACAGCAAATGGGTCGCGGATCCGAATTCATGCTA<br>AAAGTATTTAATCCCTCACCTGT	To<br>generate

STM4315-R	GTGGTGGTGCTCGAGTGCGGCCGC<u>AAGCTT</u>TCAATTAACATATTGATGACGAGAGGAAG	pET28a-*STM4315*
STM0109-F	GGACAGCAAATGGGTCGCGGATCC<u>GAATTC</u>ATGCCCTCAGGTCGCCTGCA	To generate
STM0109-R	GTGGTGGTGCTCGAGTGCGGCCGC<u>AAGCTT</u>TTAAGGATCTGGCGGCGCAAAC	pET28a-*STM0109*
STM0030-F	GGACAGCAAATGGGTCGCGGATCC<u>GAATTC</u>ATGCGGCCAATAAAAAATGCTAAAAAAATTGAC	To generate
STM0030-R	GTGGTGGTGCTCGAGTGCGGCCGC<u>AAGCTT</u>TTAAACCGACAAATCGCATAAGGATGTGCTTC	pET28a-*STM0030*
STM3908-F	GGACAGCAAATGGGTCGCGGATCC<u>GAATTC</u>GTGGATTTACGCGATCTAAAAACC	To generate
STM3908-R	GTGGTGGTGCTCGAGTGCGGCCGC<u>AAGCTT</u>CTAATTCGGTAAAATCTTCCAGAACG	pET28a-*STM3908*
STM0864-F	GGACAGCAAATGGGTCGCGGATCC<u>GAATTC</u>ATGGAAACGCGACGCGA	To generate
STM0864-R	GTGGTGGTGCTCGAGTGCGGCCGC<u>AAGCTT</u>ACATCAGAGTAATTTGTTGCGC	pET28a-*STM0864*
STM3543-F	GGACAGCAAATGGGTCGCGGATCC<u>GAATTC</u>ATGAAAAAGAAAAGACCCGTAC	To generate
STM3543-R	GTGGTGGTGCTCGAGTGCGGCCGC<u>AAGCTT</u>CTAAATAGATCCGCCCGGT	pET28a-*STM3543*
STM4586-F	GGACAGCAAATGGGTCGCGGATCC<u>GAATTC</u>GGATCAGGCTGGCATAATTC	To generate
STM4586-R	GTGGTGGTGCTCGAGTGCGGCCGC<u>AAGCTT</u>TTAACGGCGAATCGGGATC	pET28a-*STM4586*
STM1437-F	GGACAGCAAATGGGTCGCGGATCC<u>GAATT</u>CATGAATAAGCAAACCGAACACGA	To generate
STM1437-R	GTGGTGGTGCTCGAGTGCGGCCGC<u>AAGCTT</u>TTACTGCTCAGGCGTTGCAAT	pET28a-*STM1437*
STM2575-F	GGACAGCAAATGGGTCGCGGATCC<u>GAATTC</u>ATGAATATCAAACAACTACATTCC	To generate
STM2575-R	GTGGTGGTGCTCGAGTGCGGCCGC<u>AAGCTT</u>TTATACGCCTATTTTCTGCT	pET28a-*STM2575*
STM4125-F	GGACAGCAAATGGGTCGCGGATCC<u>GAATTC</u>ATGAATATTCGTGATCTTGAATA	To generate
STM4125-R	GTGGTGGTGCTCGAGTGCGGCCGC<u>AAGCTT</u>TTAAACCGCCTGTTTTAAC	pET28a-*STM4125*
STM1315-F	GGACAGCAAATGGGTCGCGGATCC<u>GAATTC</u>ATGATGCAGCTACAGGTTAACGC	To generate
STM1315-R	GTGGTGGTGCTCGAGTGCGGCCGC<u>AAGCTT</u>TTACTCATTAATTTCCGTCAATCGC	pET28a-*STM1315*
STM2572-F	GGACAGCAAATGGGTCGCGGATCC<u>GAATTC</u>ATGAACTGTTTGATTCGTATTCG	To generate

STM2572-R	GTGGTGGTGCTCGAGTGCGGCCGC<u>AAGCTT</u>TCAAACCAGTTTTTTTACCAGC	pET28a-*STM2572*
STM3215-F	GGACAGCAAATGGGTCGCGGATCC<u>GAATTC</u>ATGATGCAAAATCAACATGAAGGGT	To generate
STM3215-R	GTGGTGGTGCTCGAGTGCGGCCGC<u>AAGCTT</u>TAATCCAGTTCGGCGATTTCC	pET28a-*STM3215*
STM0354-F	GGACAGCAAATGGGTCGCGGATCC<u>GAATTC</u>ATGAACATCGGTAAAGCAGCTA	To generate
STM0354-R	GTGGTGGTGCTCGAGTGCGGCCGC<u>AAGCTT</u>TACAGACGCTTTGCCAGTC	pET28a-*STM0354*
STM3175-F	GGACAGCAAATGGGTCGCGGATCC<u>GAATTC</u>ATGAATGACCTGATCAGCGC	To generate
STM3175-R	GTGGTGGTGCTCGAGTGCGGCCGC<u>AAGCTT</u>TAACGAAGCGGCAGATAGATAT	pET28a-*STM3175*
STM2265-F	GGACAGCAAATGGGTCGCGGATCC<u>GAATTC</u>ATGATGAAAAAAGCGTTACTTATC	To generate
STM2265-R	GTGGTGGTGCTCGAGTGCGGCCGC<u>AAGCTT</u>CTACTCCTCTTTTTGCGCTTC	pET28a-*STM2265*
STM3262-F	GGACAGCAAATGGGTCGCGGATCC<u>GAATTC</u>ATGAACTCATTTGAGCGAAGA	To generate
STM3262-R	GTGGTGGTGCTCGAGTGCGGCCGC<u>AAGCTT</u>TAGACGATCATTAATTCCACG	pET28a-*STM3262*
STM3245-F	GGACAGCAAATGGGTCGCGGATCC<u>GAATTC</u>ATGAATACTCTTGTTCTCCCTAA	To generate
STM3245-R	GTGGTGGTGCTCGAGTGCGGCCGC<u>AAGCTT</u>TCATTCAATTTCTATTAACTGCC	pET28a-*STM3245*
STM1704-F	GGACAGCAAATGGGTCGCGGATCC<u>GAATTC</u>ATGAACTCCCGACAACAATCAATT	To generate
STM1704-R	GTGGTGGTGCTCGAGTGCGGCCGC<u>AAGCTT</u>TAAACGGATGAGCCAATAACATTG	pET28a-*STM1704*
STM2424-F	GGACAGCAAATGGGTCGCGGATCC<u>GAATTC</u>ATGAACTATTCACTGCGTCAACT	To generate
STM2424-R	GTGGTGGTGCTCGAGTGCGGCCGC<u>AAGCTT</u>TATAGCTGATACAGCGGATCTTT	pET28a-*STM2424*
STM1588-F	GGACAGCAAATGGGTCGCGGATCC<u>GAATTC</u>ATGCCGGGTACGGAAAAAAC	To generate
STM1588-R	GTGGTGGTGCTCGAGTGCGGCCGC<u>AAGCTT</u>TACATGAAGTACTGATTTTTAATCGTCG	pET28a-*STM1588*
STM0115-F	GGACAGCAAATGGGTCGCGGATCC<u>GAATTC</u>ATGCCAGAGGTCAAAACC	To generate
STM0115-R	GTGGTGGTGCTCGAGTGCGGCCGC<u>AAGCTT</u>TATCGCTTACAAACAGAGACTA	pET28a-*STM0115*
STM4455-F	GGACAGCAAATGGGTCGCGGATCC<u>GAATTC</u>ATGCAAAACCGGCTCACTAT	To generate

STM4455-R	GTGGTGGTGCTCGAGTGCGGCCGC<u>AAGCTT</u>TCAGGC GAGGGTAGAAGGA	pET28a-*STM4455*
STM4322-F	GGACAGCAAATGGGTCGCGGATCC<u>GAATTC</u>GTGCAAC GTGAAGATATTCTGGGAG	To generate
STM4322-R	GTGGTGGTGCTCGAGTGCGGCCGC<u>AAGCTT</u>TCAGGT GAGCGCTCCACCCT	pET28a-*STM4322*
STM3357-F	GGACAGCAAATGGGTCGCGGATCC<u>GAATTC</u>GTGCAG AAGCCTAAGTTAGGGAAG	To generate
STM3357-R	GTGGTGGTGCTCGAGTGCGGCCGC<u>AAGCTT</u>TCAGG CAGAAGGGTCACCAAC	pET28a-*STM3357*
STM4481-F	GGACAGCAAATGGGTCGCGGATCC<u>GAATTC</u>ATGAGGA ATCACAGAATTTCTTTG	To generate
STM4481-R	GTGGTGGTGCTCGAGTGCGGCCGC<u>AAGCTT</u>TTATAA CGTGCTGCCGTGATA	pET28a-*STM4481*
STM4049-F	GGACAGCAAATGGGTCGCGGATCC<u>GAATTC</u>GTGGCA AATCAGTTAATCCTT	To generate
STM4049-R	GTGGTGGTGCTCGAGTGCGGCCGC<u>AAGCTT</u>TTAATC GCTTTGATTACTGAGAT	pET28a-*STM4049*
STM3736-F	GGACAGCAAATGGGTCGCGGATCC<u>GAATTC</u>ATGAAC TTGCTGCAAATGG	To generate
STM3736-R	GTGGTGGTGCTCGAGTGCGGCCGC<u>AAGCTT</u>TTATCG GTAGTACGTTTTTAATTTC	pET28a-*STM3736*
STM1127-F	GGACAGCAAATGGGTCGCGGATCC<u>GAATTC</u>ATGGAG CCTCAACCCCCAC	To generate
STM1127-R	GTGGTGGTGCTCGAGTGCGGCCGC<u>AAGCTT</u>TTATTT TGTCATGTCCAGGGCAT	pET28a-*STM1127*
STM0333-F	GGACAGCAAATGGGTCGCGGATCC<u>GAATTC</u>ATGAAAA CGACACCTGTAGGA	To generate
STM0333-R	GTGGTGGTGCTCGAGTGCGGCCGC<u>AAGCTT</u>TCACAG TACGCCCGGTG	pET28a-*STM0333*
